# Immune cell senescence in autoimmunity: implications for disease pathogenesis and therapeutic targeting

**DOI:** 10.3389/fimmu.2025.1596686

**Published:** 2025-08-07

**Authors:** Fei Yin, Yangfang He, Jian Li, Yuan Gao

**Affiliations:** ^1^ Department of Neurology, The Second Hospital of Jilin University, Changchun, China; ^2^ Department of Endocrinology and Metabolism, The Second Hospital of Jilin University, Changchun, China; ^3^ Department of Emergency and Critical Care Medicine, The Second Hospital of Jilin University, Changchun, China

**Keywords:** immune cell senescence, autoimmune disease, senescence-associated secretory phenotype, senolytics, monoclonal antibody

## Abstract

The senescence of immune cells has also emerged as a key hallmark of immunological dysregulation and chronic inflammation in autoimmunity. Senescent immune cells are irreversibly arrested in the cell cycle, exhibit antimetabolic characteristics, and secrete pro-inflammatory mediators, all together disrupting immune homeostasis. T cells, B cells, and innate immune subsets, acquire a senescence-associated secretory phenotype (SASP), which initiates tissue damage and sustains continuous inflammation in autoimmune diseases. The accumulation of senescent immune cells undermines immune surveillance, disrupts self-tolerance mechanisms, and enhances autoantibody production, all of which contribute to the pathogenesis of autoimmune diseases, including type 1 diabetes (T1D), systemic lupus erythematosus (SLE), and rheumatoid arthritis (RA). Accumulating evidence reveals that metabolic stress, chronic DNA damage, and persistent antigenic exposure in inflammatory microenvironments induce immune cell senescence. Such senescent condition more aggressively promotes disease pathogenesis by compromising antigen presentation, disrupting cytokine signaling, and weakening the function of regulatory T cells (Tregs). Targets of senolytic drugs, SASP inhibitors, monoclonal antibodies (mAbs), and CAR T cell therapy currently have the potential to accelerate autoimmune pathology. These treatments would be directed specifically against the selective elimination or reprogramming of senescent cells to restore immune homeostasis. This review examines the mechanistic relationships between autoimmune development and immune cell senescence, as well as recent advancements in senescence-directed therapy. Understanding these pathways can provide new insights into autoimmune pathogenesis and inform future therapeutic approaches to immune cell aging.

## 1 Introduction

Autoimmune diseases result from immune system dysfunction that mistakenly attacks healthy tissues, causing tissue destruction, persistent inflammation, and impaired organ function (1, 2). Although genetic susceptibility and environmental factors are well-recognized etiological contributors, cellular senescence has recently been identified as a significant driver of autoimmune pathogenesis (3, 4). This relationship is largely mediated by immunosenescence—the progressive deterioration and functional dysregulation of immune responses associated with aging (5). Importantly, immunosenescence and immune cell senescence are distinct yet interconnected processes. Immunosenescence refers to the systemic deterioration of immune responses with age, whereas immune cell senescence involves individual immune cells undergoing functional loss and permanent growth arrest {6 #318}. Although these processes are interrelated, they have distinct biological triggers and consequences.

Senescent immune cells, especially B cells, CD4+ and CD8+ T lymphocytes, and innate immune cells, such as macrophages and dendritic cells (DCs), acquire a distinctive secretory profile associated with cellular senescence. These cells persistently release inflammatory signaling molecules, such as tumor necrosis factor (TNF)-α, interleukin-6 (IL-6), interferon (IFN)-γ, and granulocyte-macrophage colony-stimulating factor (GM-CSF), creating a systemic pro-inflammatory environment characteristic of age-related chronic inflammation (1, 2).

In the context of autoimmune pathogenesis, this inflammatory milieu promotes three key pathological processes: stimulation of self-reactive lymphocytes, breakdown of peripheral immune tolerance, and functional impairment of regulatory T cell (Treg) populations (7). Notably, senescent cells frequently develop apoptotic resistance, enabling their prolonged survival and sustained inflammatory signaling. This establishes a vicious cycle wherein persistent inflammation promotes additional immune cell senescence, exacerbating autoimmune progression. Also, age-associated declines in DC antigen presentation efficiency and B cell antibody diversity further compromise immune regulation (5).

This review summarizes the dynamic association between immune cell senescence and autoimmune pathogenesis. Deciphering the molecular mechanisms of immune cell aging offers critical insights disease onset and new therapeutic opportunities. New approaches to targeted strategies, such as senolysis, cellular metabolic regulation and functional rejuvenation, have potential for the re-establishment of immunological homeostasis and prevention of autoimmune disease.

## 2 Immune cell senescence

Three key drivers accelerate immune cell senescence: failing mitochondrial energy production, erosion of protective chromosome ends (telomeres), and repeated immune activation over time (8). These aged immune cells are particularly problematic due to their resistance to apoptosis and simultaneous secretion of inflammatory signals known as senescence-associated secretory phenotype (SASP). This creates a double-edged sword—the cells persist abnormally while promoting chronic inflammation that further disrupts immune function (9).

## 3 Role of senescent immune cells in autoimmunity

**Figure 1:**
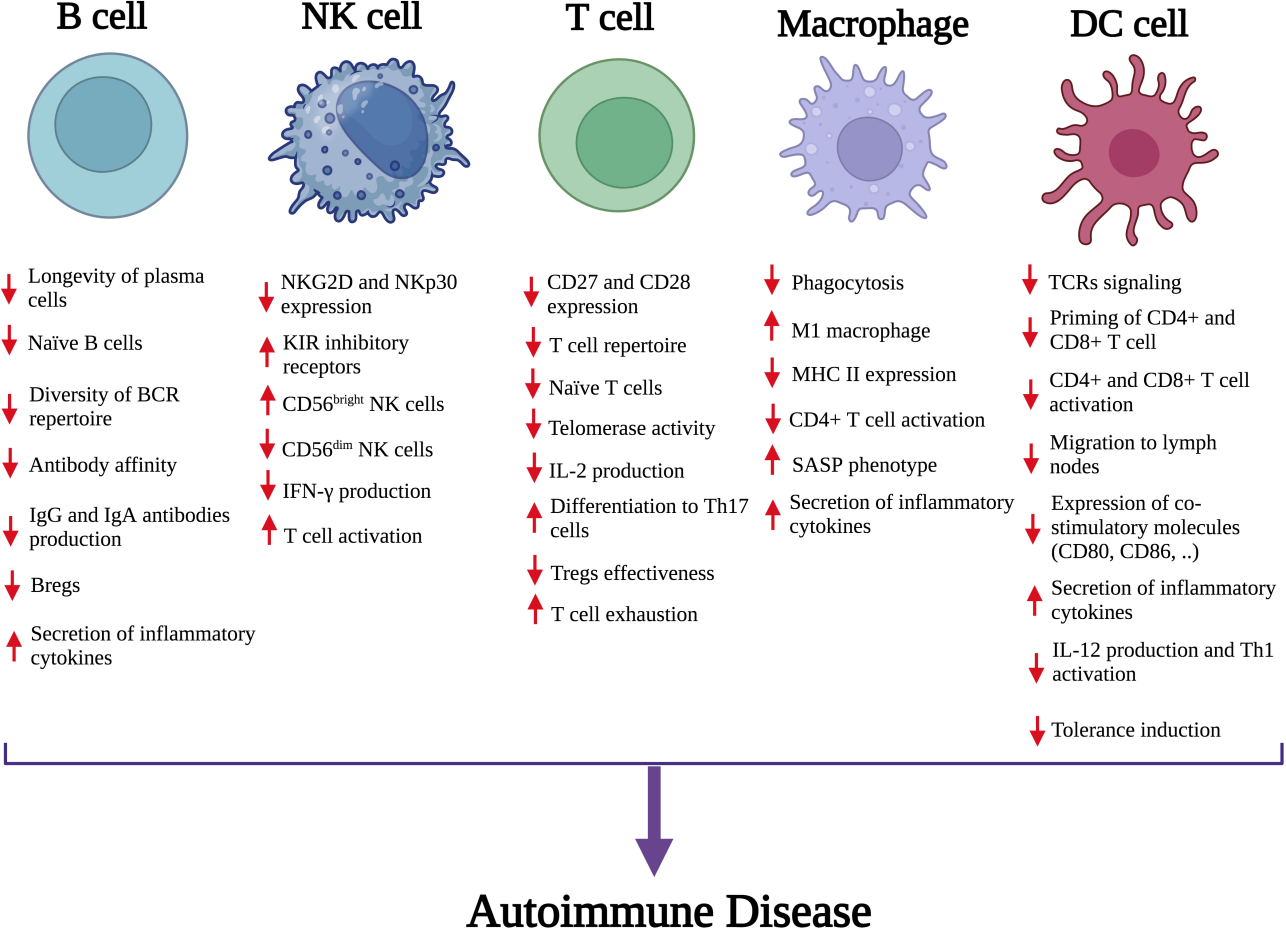
The impact of senescence on key immune cells and their contribution to autoimmune disease. Senescence leads to functional impairments in both innate and adaptive immune cells, including B cells, NK cells, T cells, macrophages, and DCs. Notable changes include impaired activation and function of immune cells, altered cell surface markers and secretion of inflammatory cytokines. These cumulative effects disrupt immune tolerance and increase inflammatory responses, promoting the development of autoimmune disorders. NK, Natural killer; DC, Dendritic cell; Breg, Regulatory B cell; BCR, B-cell receptor; IgA, Immunoglobulin A; IgG, Immunoglobulin G; NKG2D, Natural killer group 2D; CD, Cluster of differentiation; NKp30, Natural killer protein 30; KIR, Killer cell immunoglobulin-like receptors; IFN‐γ, Interferon‐gamma; IL-2, interleukin-2; Th17, T helper 17; SASP, Senescence-associated secretory phenotype; TCR, T-cell receptor; MHC II, Major histocompatibility complex.

Cellular senescence in immune cells is a key mechanism in autoimmune pathogenesis. Profiling senescent immune subsets—especially aging neutrophils, macrophages, dendritic cells, natural killer (NK) cells, B lymphocytes, and T lymphocyte subsets—is crucial in order to make them realize their pathophysiological functions. Age-related cellular changes resulting from such alterations cause: inefficient effector functions, disruption of immune homeostasis, and gradual failure of surveillance mechanisms (**Figure 1**) (10).

### 3.1 T cell senescence and autoimmunity disorders

**Figure 2:**
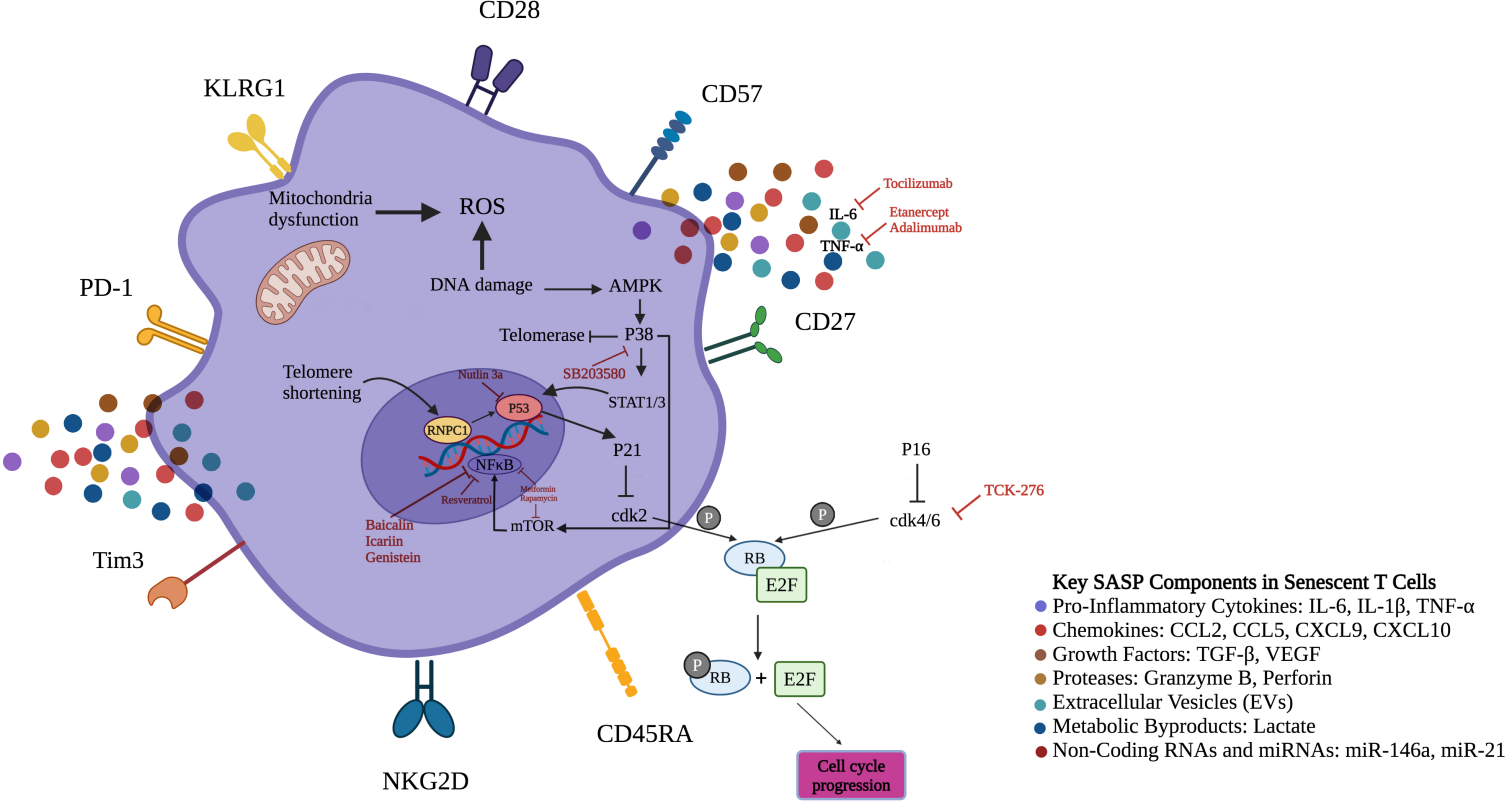
Senescent T-cells surface markers, signaling pathways and therapeutic targets. The figure illustrates the distinct expression pattern of surface markers on senescent T-cells. In contrast to normal T-cells, senescent T-cells demonstrate elevated expression levels of CD45RA, NKG2D, PD-1, CD57, Tim-3, and KLRG1, while displaying reduced levels of CD27 and CD28. Low levels of adenosine triphosphate and endogenous DNA damage activate AMPK, inducing constitutive P38 expression in senescent T-cells. Moreover, glucose deprivation and genotoxic stress, which result in P38 activation, lead to a reduction in telomerase activity and the inhibition of T-cell proliferation, representing two key features of senescence. Additionally, active P38 induces transcription factors STAT1/3 to boost levels of CKIs like P21 and P16 that halt T cell proliferation through inhibition of cdk2 and cdk4/6, respectively. P38 could also increase transcription of NF-κB by mTOR induction. Senotherapeutics inhibit senescence by suppressing SASP expression via targeting NF-κB, mTOR and P38. CD45RA, Cluster of differentiation 45 isoform R; NKG2D, Natural killer group 2D; PD-1, Programmed cell death-1; KLRG1, Killer cell lectin like receptor G1; NF-κB, Nuclear factor kappa-light-chain-enhancer of activated B cells; AMPK, Adenosine monophosphate-activated protein kinase; CKI, Cyclin-dependent kinase inhibitor; cdk2, Cyclin-dependent kinase 2; SASP, Senescence-associated secretory phenotype; mTOR, Mammalian target of rapamycin; E2F, Early region 2 binding factor; RB, Retinoblastoma tumor suppressor protein; T-cell immunoglobulin and mucin domain-containing protein 3; TNF-α, Tumor necrosis factor alpha; ROS, Reactive oxygen species; STAT, Signal transducer and activator of transcription; RNPC1, Investigated RNA-binding region-containing protein 1.

With age, T lymphocytes undergo significant phenotypic and functional changes, resulting in increased populations of senescent CD4+ and CD8+ T cells. These cells characteristically lose costimulatory receptors (CD27/CD28) and gain NK-like markers, including killer cell lectin-like receptor subfamily G member 1 (KLRG1) and CD57 (**Figure 2**) (11, 12). Senescent CD4+ T cells adopt unconventional characteristics and interact with major histocompatibility complex (MHC) class I molecules via non-classical signaling pathways. Moreover, they regulate conventional T cell signaling thresholds and boost proinflammatory responses by c-Jun N-terminal kinase (JNK) activation (13). Besides these cellular alterations, they also acquire severe metabolic dysregulation—marked by excessive secretion of cytokines (14). The overproduction of IFN-γ disrupts immune regulation through suppression of cytotoxic T-lymphocyte-associated protein 4 (CTLA-4), programmed death-ligand 1 (PD-L1), and indoleamine 2, 3-dioxygenase (IDO), weakening tolerance checkpoints (15). Additionally, glucose transporter Glut1 and fatty acid transporters FATP2/3 impairment and mitochondrial dysfunction further compromise T cell fitness (16). Sustained exposure to cytokines and DNA damage cause sustained activation of the extracellular signal-regulated kinase (ERK) and P38 mitogen-activated protein kinases (MAPKs) cascades, activate cell cycle regulators (P53, P21, P16), which enforce cell cycle arrest and telomerase suppression—hallmarks of senescence (17–19). CD28-CD8+ T cells maintain cytotoxic capacity through perforin/granzyme release, regulated by T box 21 (TBX21) and Eomes transcription factors and mammalian target of rapamycin (mTOR) signaling (20–22). The rheumatoid arthritis (RA) milieu polarizes CD4+ T cells to the pro-inflammatory T helper (Th) 17 phenotype with increased secretion of IL-17 and IL-22. In parallel, Tregs decrease in number and function, limiting their transforming growth factor-β (TGF-β) and IL-10-mediated control of immune responses. This imbalance of the Th17/Treg ratio interferes with immunological tolerance mechanisms. This dysregulation is enhanced by IL-6 to induce the generation of T follicular helper (Tfh) cells that strongly stimulate autoreactive B lymphocytes to enhance autoantibody production and tissue destruction (23, 24).

Clinical evidence strongly supports the role of senescent T cells in autoimmunity. In RA (25, 26), type 1 diabetes (T1D) (27), multiple sclerosis (MS) (28), Graves’ disease (GD) (29), and granulomatosis with polyangiitis (30), CD4+CD28- T cells are capable of infiltrating inflamed tissues and exacerbating immune-mediated damage. These types of cells may exhibit high expression levels of CX3C motif chemokine receptor 1 (CX3CR1), whose ligand fractalkine is elevated in RA synovium (31) and cerebrospinal fluid (CSF) of patients with MS (32). Telomere shortening, another hallmark of senescence, occurs prematurely in both naïve and memory T cells in RA patients—even as early as in their twenties (33, 34). In systemic lupus erythematosus (SLE), senescent CD8+ and CD57+ T cells are associated with disease severity and anemia (35). Together, these findings underscore the central role of T cell senescence in the breakdown of immune tolerance and the amplification of autoimmune inflammation. Targeting the pathways that drive T cell senescence, including metabolic stress, SASP signaling, and checkpoint dysregulation, holds promise for therapeutic intervention in a range of autoimmune diseases.

### 3.2 B cell senescence and autoimmunity disorders

**Figure 3:**
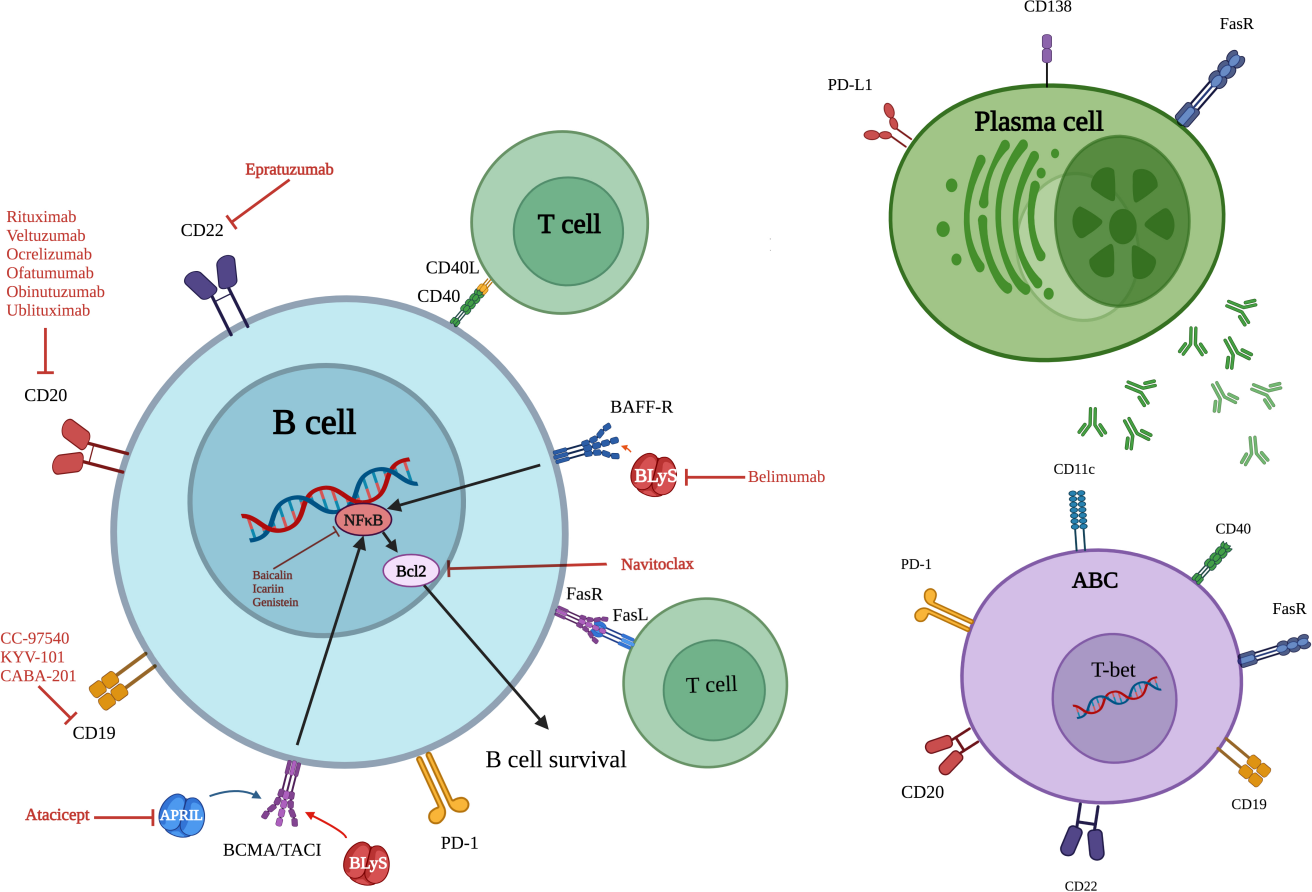
Overview of key surface markers, signaling pathways and therapeutic targets of senescent B cells, ABCs and plasma cells. ABC, Age associated B cell; CD, Cluster of differentiation; BAFF-R, B cell activating factor receptor; TACI, Transmembrane activator and calcium modulator and cyclophilin ligand (CAML) interactor; BLyS, B lymphocyte stimulator; APRIL, A proliferation-inducing ligand; FasL (Fas ligand or CD95L); FasR, Fas receptor; PD-1, Programmed cell death protein; PD-L1, Programmed death-ligand 1; T-bet, T-box expressed in T cells; Bcl-2, B-cell lymphoma/lymphoma 2; BCMA, B-cell maturation antigen; NF-κB, Nuclear factor kappa-light-chain-enhancer of activated B cells.

While B lymphocytes are essential mediators of antibody-mediated immunity, their functional and phenotypic characteristics undergo substantial age-related modifications (36). A particularly significant alteration is the progressive expansion of a specialized B cell population—termed age-associated B cells (ABCs)—characterized by surface expression of CD11c and the transcription factor T-bet, along with downregulation of CD21 and CD23 markers (37, 38). Unlike conventional B cell activation through antigen receptor engagement, these atypical cells primarily develop in response to endosomal pattern recognition receptors (toll-like receptor (TLR)-7 and TLR-9 stimulation). Their prevalence escalates with advancing age and is notably elevated in autoimmune pathologies (39). Functionally, ABCs demonstrate both autoreactive potential and enhanced antibody secretory capacity (40). The aging process disrupts the delicate equilibrium of transcriptional regulators governing B cell biology. Critical factors including E2A (41) and paired box 5 (PAX5) (42), which normally preserve B cell identity and repertoire diversity, demonstrate diminished expression (**Figure 3**). This transcriptional dysregulation promotes autoreactive tendencies while compromising the capacity to mount responses against new antigens. Furthermore, aging substantially reduces expression of X box binding protein-1 (XBP-1) and B lymphocyte inducer of maturation program 1 (Blimp-1) in B-1 cells—master regulators of antibody production—as evidenced in studies of elderly populations (43). Aged B cells exhibit extensive metabolic changes, including mitochondrial impairment and increased proinflammatory capacity. The senescent cells acquire a typical secretory profile that is associated with increased levels of IL-10, IL-6, and TNF-α. Such cytokine secretion maintains inflammatory microenvironments and further impairs Tfh regulation, eventually impeding key germinal center function such as antibody affinity maturation and isotype switching (44). Circulating inflammatory mediators, particularly those originating from adipose tissue depots, can promote senescence acquisition in peripheral B cell populations. This mechanism establishes a direct connection between metabolic dysregulation and age-related immune dysfunction, linking pro-inflammatory adipokines to accelerated immune aging (45, 46). The functional alterations in aged B lymphocytes play a direct role in autoimmune disease development. ABCs show increased propensity to develop into self-reactive antibody producers, especially when exposed to IL-21 and IFN-γ microenvironments that override normal tolerance mechanisms. Concurrently, regulatory B cell populations experience both numerical reduction and functional decline, diminishing their critical immunosuppressive functions mediated through IL-10 and -35 secretion (47). The growing imbalance between pro-inflammatory ABCs and diminishing regulatory B cell (Breg) populations erodes critical immune tolerance mechanisms. This disequilibrium fosters persistent inflammatory states characteristic of RA and SLE pathogenesis (11).

Multiple clinical investigations reveal significant expansions of senescent B cell populations across autoimmune disorders. Patients with SLE demonstrate elevated levels of CD19+CD11c+T-bet+ ABCs, which show positive correlation with both autoantibody titers and clinical disease severity. RA cases similarly display increased circulating CD95+ activated memory B cells that participate in sustained joint inflammation. During acute lupus flares, the continued presence of autoreactive naïve B cell populations highlights profound regulatory dysfunction. Experimental models further demonstrate that CD19+CD138+ B cell subsets play pivotal roles in driving neuroinflammation in the lymph nodes of mice with induced autoimmune encephalomyelitis (48). Patients with RA consistently demonstrate elevated peripheral blood levels of CD95+ activated memory B cells (23). These clinical observations underscore the pathogenic role of B cell senescence in autoimmune pathogenesis and reveal promising opportunities for therapeutic intervention through senescent B cell modulation.

### 3.3 NK cell senescence and autoimmunity

NK cells serve as critical effectors of innate immunity, specializing in the rapid detection and destruction of virally infected and cancerous cells without requiring prior antigen exposure. These lymphocytes develop directly from hematopoietic stem cells through a thymus-independent differentiation pathway, distinguishing them from adaptive T lymphocytes (49). Human NK cells are broadly categorized into two functionally distinct subsets according to CD56 surface density: The CD56^bright^ population demonstrates superior cytokine-secreting capacity and predominates during developmental stages, while CD56^dim^ cells exhibit enhanced cytotoxic potential and progressively increase with aging to become the major circulating subset (50). With increasing age, NK cell populations experience marked functional and phenotypic alterations. Although their absolute numbers are augmented, competency is compromised by diminished expression of cytokine receptor (IL-2R, IL-15R, IL-21R) and reduced sensitivity to these essential survival cues (51). Senescent features—such as restricted proliferative capability, reduced cytotoxic function, and deregulated cytokine production—characterize aged NK cells. While less well explored than lymphocyte senescence, senescence of NK cells also contributes to immune dysfunction and can entail the gain of a pro-inflammatory secretory phenotype, marked by increased TNF-α and IFN-γ release. Although these cytokines provide protective mechanisms, chronic overproduction promotes inflammatory tissue pathology. Other age-related impairments—including reduced receptor repertoire diversity, signaling competence, and degranulation capacity—collectively limit NK cell-mediated immune surveillance in the elderly (52). Aged NK cells play a significant role in autoimmune disease development through their impaired ability to maintain immune equilibrium. A striking example occurs in RA, where aging CD4+ T cells lacking CD28 expression aberrantly acquire NK cell characteristics, including CD161 and KIRs. These transformed T cells develop cytotoxic capabilities resembling innate immune effectors and actively migrate to synovial tissue, where they perpetuate inflammatory damage (53). RA patients developing vascular manifestations (e.g., vasculitis) demonstrate increased co-expression of the activating receptor KIR2DS2 with its HLA-C ligand (54). The NKG2D receptor similarly contributes to autoimmune pathology in both RA and T1D by amplifying cytotoxic effector functions that drive tissue injury (55, 56). Clinical evidence further underscores NK cell involvement in autoimmunity—SLE patients show significant NK cell depletion in peripheral blood, which strongly associates with elevated IFN-α levels and increased disease severity (57, 58). While numerically decreased in SLE, NK cells display abnormal functional hyperactivity characterized by excessive IFN-γ production. This cytokine overexpression drives autoimmune amplification, as evidenced by murine models where sustained IFN-γ exposure precipitates lupus-like pathology (59). In aging individuals, the loss of balance between NK cell cytotoxic activity and immunoregulatory capacity emerges as a key contributor to failed self-tolerance and sustained inflammatory autoimmunity (60).

### 3.4 Macrophages and neutrophil senescence in autoimmunity disorders

Innate immune cells, including monocytes, macrophages, and neutrophils play crucial roles in the promotion of inflammation and immunomodulation (61). These groups undergo considerable functional changes during aging that impair host defense while enhancing chronic low-grade inflammation (62–64). Of particular interest is the age-related expansion of in proinflammatory monocyte subsets (CD14+CD16+ intermediate and CD14^dim^CD16^bright^ non-classical) bearing characteristics of cellular senescence such as telomere shortening and constitutive cytokine production (65, 66). Non-classical monocytes also elevate chemokine receptors that encourage them to be recruited into regions of inflammation (67). Aged macrophages favor proinflammatory M1 polarization phenotypes elicited by enhanced production of IL-8 and TNF-α (68). Aged neutrophils also exhibit enhanced survival, enhanced oxidative burst, and greater expression of cell surface-activating Fcγ receptors (69). In young NOD mice, active infiltration of innate and adaptive immune cells—especially neutrophils, macrophages, plasmacytoid DCs, and B lymphocytes—into islet tissue is caused by killing of pancreatic β cells (70). Senescent aged immune cells accumulate a typical secretory profile with increased release of inflammatory mediators (IL-1β, TNF-α, IL-6, IL-8) and chemokines. In addition, aging greatly impairs monocyte function by three main deficits: compromised TLR signaling responses, reduced MHC class II presentation, and dysregulation of interferon production (IFN-γ, IFN-α), which are required for antiviral defense (71). Epigenetic remodeling in aged monocytes, such as modified DNA methylation patterns, histone reorganization, and disruption of transcription factor signaling—IRF, forkhead box protein P3 (FOXP3), nuclear factor kappa-light-chain-enhancer of activated B cells (NF-κB), and signal transducer and activator of transcription (STAT)—causes significant changes in inflammatory gene expression (72). This molecular reprogramming contributes to defective production of key immune mediators (IFN-γ, IFN-α, chemokine C-C motif ligand (CCL) 20, IL-1β and CCL8), impairing antiviral immunity and leukocyte recruitment (73). Senescent macrophages also have activated TAM receptor kinases (Axl, Mer, Tyro3) that suppress innate immune activation by inhibitory signaling (74). SASP in T1D increases pathologic reactive oxygen species (ROS) production, cellular acidification, and proinflammatory M1 polarization through sustained NF-κB activation (75). Pathological events in MS also involve senescent microglia that acquire an impaired ability to clear cellular debris while acquiring a neurotoxic secretory phenotype. The subsequent accumulation of myelin breakdown byproducts is detrimental to the maturation of oligodendrocyte precursors, and oxidative stress causes mitochondrial dysfunction and axonal degeneration (76). Neutrophils that age also display marked metabolic changes such as glycolytic dependence, diminished phagocytosis, and pathologic NETosis—all of which contribute to autoimmune disease and tissue damage (77). Senescent abnormal NK cells participate in autoimmune disease pathogenesis by various mechanisms. Hyperproduction of TNF-α and IL-1β by proinflammatory M1 macrophages with increased expression of CXCR3, CCR5, and CCR8 chemokine receptors supports T1D-induced killing of pancreatic β-cells (78). RA is a result of a vicious cycle in which senescent synovial fibroblasts produce inflammatory mediators causing recruitment of macrophages and their M1 polarization with increased IL-8 and TNF-α production (68). RA neutrophils play a role in perpetuating joint injury through three such key pathological mechanisms: impaired apoptotic clearance, excessive oxidative burst activity, and dysregulated neutrophil extracellular trap formation (69, 77). Aberrant apoptosis and metabolic dysfunction distinctly influence autoimmune pathogenesis. SLE exhibits enhanced apoptotic clearance that promotes autoantigen exposure and subsequent autoantibody generation. Conversely, RA demonstrates impaired apoptotic pathways that prolong inflammatory cell survival and sustain tissue-damaging enzyme release (69, 77). Metabolically reprogrammed neutrophils in both disorders display increased glycolytic flux coupled with diminished antioxidant capacity, resulting in pathological oxidative stress that amplifies immune activation (77). Notably, the frequent co-occurrence of atherosclerosis in SLE patients stems from proinflammatory M1 macrophage activity, evidenced by elevated circulating levels of TNF-α, IFN-γ, IL-6 and IL-12—establishing a direct connection between immunosenescence and cardiovascular risk (69).

## 4 Therapeutic implications for targeting senescent immune cells in autoimmune diseases

Current therapeutic strategies in autoimmune diseases often lack specificity. Restoring immune balance by targeting senescent immune cells is of particular interest, where complex networks of aged immune cells converge to drive autoimmune pathology. Precision modulation of these molecular pathways is employed by current emerging therapies. The ensuing review integrates preclinical and clinical data from model systems in the context of devising novel approaches for understanding and intervening in the role of aged immune cells in autoimmune pathophysiology.

### 4.1 Targeting cellular senescence with senotherapeutics

Emerging senotherapeutic agents represent a novel class of small-molecule interventions designed to counteract cellular senescence in age-related immune disorders. These compounds operate through two distinct mechanisms: senomorphic agents, which modulate the SASP to reestablish immune equilibrium, and senolytic drugs, which preferentially clear senescent cell populations (**Table 1**). Such targeted approaches offer the potential to disrupt the chronic inflammatory cycles and restore immune function in autoimmune pathogenesis.

**Table 1:** Senolytics and senomorphics and their mechanisms of action on autoimmune diseases.

Class	Compound	Mechanism	Autoimmune disease
Senolytics			
Tyrosine kinase inhibitor	Dasatinib	-Inhibits pathways that support survival of senescent cells	RA (79)
Bcl-2 family inhibitor	ABT-263 (Navitoclax)	-Inhibits Bcl-2, Bcl-XL and Bcl-W -Activates the pro-apoptotic pathways	MS (80), SLE (81)
	ABT-737	-Inhibits Bcl-2, Bcl-XL and Bcl-W -Activates the pro-apoptotic pathways	RA (82), SLE (82), CLE (83)
HSP90 inhibitor	17-DMAG (Alvespimycin)	-Inhibits HSP90 -Increase in CD8+ T cells -Inducing apoptosis in senescent cells - Reduces follicular B cells	SLE (84)
	17-AAG (Tanespimycin)	-Inhibits HSP90 -Degrades the proteins involved in cell proliferation and survival	Lupus (85), Epidermolysis bullosa acquisita (86)
	Geldanamycin	-Inhibits HSP90 -Degrades the proteins involved in cell proliferation and survival	RA (87, 88)
	Ganetespib (STA-9090)	-Inhibits HSP90 -Disrupts PI3K/Akt/NF-κB, Raf/MEK/ERK and JAK/STAT3 pathways	SLE (89, 90)
	SNX-7081	-Inhibits HSP90 -Disrupts PI3K/Akt/NF-κB and Raf/MEK/ERK pathways	RA (91)
P53 Modulators	Nutlin-3a	-Inhibits P53/MDM2 interaction	RA (92), SLE (93)
Natural Compounds	Quercetin	-Inhibits anti-apoptotic pathways in senescent cells, PI3K and Bcl-2 family	T1D (94), RA (95–97), Colitis (98)
	Fisetin	-Inhibits PI3K/Akt and Bcl-2 family	SLE (99)
	Baicalin	-Inhibits NF-κB, MAPK, STAT3	T1D (100, 101), RA (102)
	Icariin	-Inhibits NF-κB, MAPK, JAK/STAT3, and NLRP3 inflammasome	T1D (103), MS (104)
	Genistein	-Inhibits NF-κB, MAPK and JAK/STAT3 -Regulates cytokine production -Promotes Treg cells	T1D (103), RA (105)
	Epigallocatechin-3-gallate	-Inhibits NF-κB, MAPK and JAK/STAT3 -Suppresses Th1 and Th17 -Activates Nrf2 -Modulates B cell proliferation	SLE (106), Psoriasis (107)
PPAR-α Agonists	Fenofibrate	-Inhibits inflammatory responses and IFN-γ and promotes IL-4 secretion	MS (108), Psoriasis (109)
	Gemfibrozil	-Inhibits NF-κB -Inhibits Th1/Th17 response -Modulates microglia and astrocytes	T1D (110), MS (111)
Senomorphics			
	Rapamycin	-Blocks mTOR, Nrf2, NF-κB	T1D (112, 113), RA (114, 115), SLE (116), JIA (117), Sjögren syndrome (118)
	Metformin	-Activates AMPK, PI3K/Akt -Inhibits mTOR, NF-κB	T1D (119), RA (120), MS (121)
	Resveratrol	-Inhibits NF-κB -Inhibits Th17 activity	IBD (122), Crohn's disease (123)
	Aspirin	-Inhibits the differentiation of naive T cells into Th17 and Th1 cells -Restores Tregs	RA (124), MS (125), SLE (126)
P38 MAP Kinase Inhibitor	SB203580	-Inhibits P38 MAP Kinase	MS (127), Lupus (128)
	Tim-3	-Inhibits P38 MAP Kinase	RA (129), Autoimmune hepatitis (130)
JAK/STAT Inhibitor	Ruxolitinib	-Inhibits JAK/STAT and decreases the proportion of Th17 cells	CLE (131), Psoriasis (132)
	Baricitinib	-Inhibits JAK/STAT pathway	MS (133), Psoriasis (134)
Cdk7 Inhibitor	BS-181	-Inhibits IL-1β, IL-6, IL-8, and RANKL via NF-κB suppression	RA (135–137)

#### 4.1.1 Development of senolytics

Cellular senescence triggers resistance to apoptosis through several pro-survival mechanisms, such as phosphoinositide 3-kinase (PI3K)/protein kinase B (Akt) pathway, B-cell lymphoma 2 (Bcl-2) protein networks, and cell cycle regulators (P21, P53, FOXO4). Senolytic drugs interfere with these protective pathways by selectively targeting these molecular protectants, as illustrated in preclinical autoimmune models (**Table 1**). This specificity allows for the elimination of pathologic senescent immune cells without destroying healthy cells (138).

##### 4.1.1.1 Dasatinib and quercetin

Dasatinib and quercetin (D+Q) senolytic combination drug is among the most promising therapeutic agents for senescent cell therapy. Dasatinib, an approved Food and Drug Administration (FDA) tyrosine kinase inhibitor, triggers apoptotic cell death in senescent cells through multi-pathway inhibition of pro-survival cues. Experimental evidence shows its ability to suppress Treg expansion by CTLA-4, FOXP3, and glucocorticoid-induced TNF receptor (GITR) downregulation, and arrest cell cycle progression in culture cells (139). Preclinical studies demonstrate dasatinib’s therapeutic potential across autoimmune models. In RA, treatment reestablished the Treg/Th17 equilibrium while ameliorating clinical disease manifestations (140). The compound similarly improved outcomes in experimental autoimmune encephalomyelitis (EAE) through dual mechanisms: suppression of microglial/macrophage activation with consequent reduction in TNF-α and matrix metalloproteinase (MMP)-2 production, and limitation of inflammatory cell central nervous system (CNS) infiltration (**Table 1**) (141).

Quercetin, a plant flavonoid with dual senotherapeutic effects, regulates important cell pathways such as PI3K and Bcl-2 signaling cascades (142). Treatment with quercetin in murine lupus models was associated with kidney protection through the downregulation of inflammatory markers (TGF-β1, TNF-α, Bcl-2-associated protein x (Bax), IL-6) and counts of senescent T cell and follicular helper T cell subsets (143). The medication also reduced pathology of RA through neutrophil recruitment inhibition and various pro-inflammatory cytokines (monocyte chemotactic protein (MCP)-1, IFN-γ, TNF-α, IL-17, IL-6) (144). Notably, in senescent macrophage-induced experimental colitis, dietary quercetin supplementation enhanced disease conditions by re-establishing heme oxygenase (HO-1)-dependent macrophage function—reinstating their anti-inflammatory potential without compromising antimicrobial function (**Table 1**) (95–97). Such broad-spectrum immunomodulation indicates that quercetin can restore mucosal immune homeostasis through site-specific macrophage reprogramming (98).

##### 4.1.1.2 Bcl-2 family inhibitors

Senescent cells tend to overexpress anti-apoptotic Bcl-2 family members (Bcl-xL, Bcl-2), making them more refractory to normal clearance pathways (145). In SLE, IL-15-induced upregulation of these survival proteins in CD4+ T cells drives the proliferation of dysfunctional lymphocytes that perpetuate inflammatory loops. Preclinical interventions that take advantage of this vulnerability have been found to be promising (**Table 1**). The senolytic drug ABT-263 (Navitoclax), which effectively depletes senescent CD4+ T cells, senescent B cells (CD19+CD11c+T-bet+), and Tfh cells, significantly reduced these populations in lupus-prone MRL/lpr mice. The mechanisms involved were accompanied by improvements in renal function, decreased proteinuria, and modulation of disease overall, establishing Bcl-2 inhibition as a valid means of autoimmune regulation (81). Therapeutic actions of senolytics on Bcl-2 are highly context-dependent across autoimmune diseases. Though therapeutic in lupus models, ABT-263 worsens disease in EAE through elimination of protective senescent microglia and macrophages—highlighting the requirement for cell-type specificity and narrow treatment windows (80). The more potent inhibitor ABT-737 (which targets Bcl-2, Bcl-xL, and Bcl-w) was employed for the treatment of SLE by inhibiting the proliferation of lymphocytes that drives disease (82). Most notably, localized ABT-737 delivery in murine cutaneous lupus models preferentially killed senescent immune infiltrates within lupus lesions (83). These findings overall indicate that whereas Bcl-2 family inhibition is beneficial, extreme caution must be exercised with regard to the tissue microenvironment and specific immune targets.

##### 4.1.1.3 HSP90 inhibitor

Heat shock proteins (HSPs), and HSP90 in particular, are essential molecular chaperones for protein folding and stability. Recent evidence indicates that they play a key role in immune cells aging and autoimmune pathogenesis. Senescent immune cells have the unique characteristic of being prone to overexpressing HSP90 as an adaptive form of cytoprotection, enhancing resistance to apoptosis and promoting pathologic persistence (146). HSP90 induces immune cell survival via senescence by stabilizing critical regulators of apoptosis. HSP90 chaperone protein maintains the structural integrity and activity of essential signaling molecules such as Bcl-2 anti-apoptotic proteins, Akt survival kinases, and NF-κB transcription factors—all generally overexpressed in old lymphocytes. Through preventing degradation of client proteins, HSP90 re-establishes pro-survival signaling networks that define senescent immune populations (147). Autoantibodies and autoreactive T cells targeting HSPs have been reported in a number of autoimmune diseases, such as MS (148), inflammatory bowel disease (IBD) (149), SLE and RA (146), denoting a pathological role for these stress proteins in breaking immune tolerance. Clinical investigations identify the increased expression of HSP90 in renal tissue and also in the circulation of SLE patients, where it promotes the survival of senescent CD4+ T cells and ABCs selectively. These senescent lymphocytes produce IL-6 as part of their SASP, generating a pro-inflammatory microenvironment that supports three pathologic pathways of especial significance: paracrine senescence transmission to adjacent immune cells, abnormal B lymphocyte activation, and autoantibody overproduction. Importantly, IL-6 also upregulates HSP90 expression, creating a self-perpetuating inflammatory loop that abrogates immune regulation and maintains disease activity in SLE (150). Pharmacologic HSP90 inhibition has been an attractive senotherapeutic option. Geldanamycin analogues, 17-DMAG (alvespimycin), and 17-AAG (tanespimycin) are cytotoxic to senescent immune subsets with specificity (**Table 1**). Initial findings prove that 17-DMAG specifically kills disease-causing lymphocyte populations in lupus models such as follicular B cells and double-negative T cells (CD4-CD8-), thus improving immune imbalance in aging models (84). Mechanistically, HSP90 drives senescence via amplification of pro-inflammatory signal transduction pathways. The preclinical agent SNX-7081 suppressed RA pathology in animal models by disrupting NF-κB activation and resulting SASP-associated cytokine production (TNF-α, IL-17, IL-6, IL-8), leading to the suppression of joint inflammation (**Table 1**) (91).

##### 4.1.1.4 P53 modulators

Therapeutic modulation of p53 signaling pathways is a new treatment strategy for immune senescence in autoimmunity. Being the master guardian of genomic integrity and cell fate choice, p53 has multi-dimensional immunomodulatory roles—maintaining immune homeostasis at basal states but causing lymphocyte dysfunction under conditions of chronic inflammation. In autoimmune pathogenesis, dysregulation of p53 facilitates pathological persistence of immune cells via two mechanisms: disrupted apoptosis signaling and ectopic activation of survival pathways, thus perpetuating inflammatory loops (151). A number of studies have also detected anti-p53 autoantibodies in a number of autoimmune diseases, such as SLE, autoimmune hepatitis, granulomatosis, and GD. Such observations suggest generalized immune recognition of structurally or functionally aberrant p53 proteins in a variety of autoimmune pathologies. Such a pathway would be likely to underlie two related disease mechanisms: senescent immune cell accumulation with aberrant p53 signaling, and subsequent loss of immune tolerance to this essential tumor suppressor protein. All together, these mechanisms form a self-reinforcing feedforward loop that maintains autoimmune activity by initiating chronic inflammatory signaling and defective apoptotic elimination of pathogenic lymphocytes (152). Increased p53 protein and certain genetic mutations (at positions 213 and 239) have been found in RA synovium, and these are associated with increased IL-6 release—a key driver of senescence-associated inflammation (153). This finding has generated therapeutic promise for p53-modulating strategies. Of new directions, the murine double minute 2 (MDM2) inhibitor Nutlin-3a is of special interest by inhibiting p53 ubiquitination and proteasomal degradation and thus augmenting its tumor suppressor activity (154). Preclinical studies show that Nutlin-3a can selectively eliminate senescent cells that are dependent on aberrant p53 pathways for survival. In models of arthritis induced by collagen, treatment with this MDM2 inhibitor elicited three major therapeutic effects: inhibition of pro-inflammatory cytokine networks, reduction of clinical arthritis scores, and inhibition of synovial inflammation. These studies unveil its dual potential to deplete simultaneously pathogenic senescent immune subsets and reduce SASP-driven tissue damage (92). The MDM2/p53 regulatory axis shows particular dysregulation in pediatric SLE, where elevated MDM2 activity drives pathological mesangial cell proliferation and disrupts normal B cell compartmentalization (93). Nutlin-3a demonstrates therapeutic potential by specifically interrupting this pathogenic signaling cascade. This class of p53-stabilizing compounds offers precise targeting of senescent immune populations while sparing healthy cells, positioning them as promising candidates for senolytic therapy (**Table 1**).

##### 4.1.1.5 Natural compounds

Plant-derived senotherapeutic agents like fisetin have been proposed as potential modulators of age-related immune dysfunction modulators in autoimmunity. Fisetin and other bioactive molecules selectively target senescent immune subsets and inhibit pro-inflammatory secretory profiles, possibly halting the vicious cycles perpetuating chronic inflammation (155). In lupus-prone MRL/lpr mice, fisetin administration produced three key therapeutic benefits: (1) amelioration of renal fibrosis, (2) clearance of senescent T lymphocytes, and (3) downregulation of senescence-associated inflammatory mediators (99). These preclinical results establish fisetin as a multifaceted senolytic agent with potential to influence autoimmune disease progression (**Table 1**).

Emerging research reveals multiple plant-derived compounds with senotherapeutic properties across autoimmune conditions, underscoring the central role of cellular senescence in pathogenesis (**Table 1**). The soy isoflavone genistein demonstrated dual metabolic and immunomodulatory benefits in T1D models, likely mediated through modulation of SASP components (100). The bioactive flavonoid icariin, derived from Epimedium species, exhibited comparable therapeutic effects in rodent models through two primary mechanisms: potentiation of cellular antioxidant defenses and suppression of NF-κB-mediated inflammatory signaling. This dual action effectively modulates the SASP by targeting its central transcriptional regulator (103). Baicalin (101), and baicalein (156), flavonoids, have strong anti-inflammatory and neuroprotective properties in model systems. Baicalein maintained motor function and decreased demyelination in EAE mice, documenting to reverse neuroinflammation mediated by senescent immune cells. Expanding on these results, a double-blind, placebo-controlled trial showed that EGCG, a green tea polyphenol, improved remission in IBD patients (157). Although its direct senolytic action on human beings is a yet-to-be-established fact, EGCG’s immunomodulatory effects has a potential in inhibiting immune senescence.

##### 4.1.1.6 PPAR-α agonists

Recent investigations reveal unexpected immunomodulatory properties of peroxisome Proliferator-Activated Receptor (PPAR)-α activating compounds, expanding their therapeutic potential beyond metabolic regulation. The fibrate class of drugs—particularly gemfibrozil, ciprofibrate and fenofibrate—demonstrates significant capacity to modify immune responses in multiple sclerosis and related conditions (**Table 1**) (158). These effects appear particularly relevant to inflammation driven by aging immune cells. Experimental data show ciprofibrate acts to regulate central elements of neuroinflammation, such as suppression of autoreactive T cell clones and regulation of overactivated microglia. Therapy is associated with typical changes in cytokine release profiles, e.g., reduced IFN-γ and increased IL-4 levels, indicating a switch towards Th2-mediated response. Simultaneous studies show that fenofibrate is capable of suppressing activation of the Th17 pathway, whereas gemfibrozil shows more generalized immunomodulation within human lymphocyte subsets (108). These collective actions position PPAR-α agonists as potential modifiers of immune aging processes in autoimmune pathogenesis.

##### 4.1.1.7 Cdks inhibitors

Cyclin-dependent kinases (Cdks), crucial regulators of cell division and gene expression, have gained attention as potential therapeutic targets for autoimmune disorders due to their role in senescence pathways. In autoimmune conditions, aberrant cell cycle control promotes premature aging of immune cells—particularly T lymphocytes—resulting in chronic inflammatory states and impaired immune regulation (159). Pharmacological modulation of Cdks offers a strategic approach to interrupt two key pathological processes in autoimmunity: the progressive accumulation of senescent immune cells and their deleterious secretory profile. By targeting Cdk activity, therapeutic interventions could break the self-perpetuating cycle of inflammation and tissue damage characteristic of chronic autoimmune conditions.

The Cdk4/6-RB-E2F axis plays a pivotal role in cell cycle regulation through phosphorylation-mediated release of transcriptional activators. In autoimmune pathogenesis, pharmacological inhibition of this pathway (e.g., via compounds such as TCK-276) may constrain pathological immune cell expansion. Preclinical arthritis models demonstrate TCK-276’s therapeutic potential, with treatment yielding both clinical improvement (reduced articular inflammation) and histological preservation (decreased joint destruction)—likely through selective suppression of proliferative, senescent-like immune populations (160, 161). At the molecular level, Cdk4/6 inhibitors functionally mimic endogenous cell cycle regulators P16 and P21, which serve as both biomarkers and critical controllers of senescence programs. Interestingly, RA synovial tissue exhibits reduced P21 expression compared with osteoarthritic joints. Experimental restoration of P21 via adenoviral vectors potently suppresses two key mediators of senescence-associated inflammation: IL-6 secretion and matrix metalloproteinase-1 production (162–164). Translational research has confirmed the clinical potential of Cdk4/6 modulation in the treatment of autoimmune diseases. A phase 1b randomized controlled trial evaluating TCK-276 in patients with RA met its primary endpoint, demonstrating statistically significant improvements in disease activity compared with placebo. These findings link preclinical mechanisms to human treatment outcomes and support Cdk inhibition as a viable strategy for immune modulation in active disease states (**Supplementary Table 1**, **Figure 2**) (165). Emerging research highlights Cdk7 as a novel regulator of inflammatory T cell responses in psoriasis. Patients show increased Cdk7 activity in circulating CD4+ T cells, with expression levels tracking closely with symptom severity. Research reveals that disrupting Cdk7 signaling—through genetic approaches or small molecule inhibitors—attenuates disease development in mouse models of psoriasis-like inflammation. This therapeutic effect coincides with reduced production of inflammatory signaling molecules (**Table 1**) (166). Collectively, pharmacological Cdk inhibitors represent a promising class of senescence-targeting therapeutics with dual mechanisms of action: direct regulation of immune cell proliferation and mitigation of senescence-driven inflammatory cascades. Their capacity to address both cell cycle dysregulation and pathological secretory phenotypes position them as versatile candidates for treating autoimmune conditions characterized by accelerated immune cell aging.

#### 4.1.2 Development of senomorphics

An alternative pharmaceutical approach focuses on xenomorphic compounds that counteract the deleterious effects of aging-related secretions while preserving cell viability. These agents prevent both the induction of aging and the production of inflammatory mediators through multiple pathways, including NF-κB, mTOR, IL-1α, and P38 MAPK signaling. Current research has identified several promising xenomorphic candidates with applications in the management of autoimmune diseases (**Table 1**).

##### 4.1.2.1 Rapamycin

The macrolide compound rapamycin demonstrates dual therapeutic properties as both an immunomodulator and senescence pathway regulator. By selectively inhibiting mTOR signaling, this clinically validated agent simultaneously suppresses immune cell activation and attenuates senescence-associated inflammatory responses, offering a unique pharmacological approach to age-related immune dysfunction (167). Rapamycin exerts its therapeutic effects primarily through mTOR pathway suppression, targeting a master regulator of cellular metabolism that becomes pathologically overactive in aged immune cells. This inhibition leads to significant downregulation of characteristic SASP—particularly the pro-inflammatory cytokines—that play fundamental roles in autoimmune disease progression (168–170). This functional reprogramming preserves cellular viability while fundamentally altering secretory behavior—transforming pro-inflammatory immune cells into more regulated phenotypes that contribute to immune homeostasis rather than perpetuate inflammatory cycles (167). Notably, rapamycin enhances autophagic flux—a critical cellular recycling process that becomes deficient in aged immune cells. This restoration of protein and organelle turnover helps maintain proper immune cell function and metabolic homeostasis, addressing a fundamental defect in senescent cell physiology (171).

Rapamycin’s immunomodulatory action is also seen in senescent cell populations in autoimmune settings. In EAE models, treatment concurrently diminished CNS infiltration of inflammatory IL-17+ T cells and boosted the percentage of Tregs—mitigating age-associated Treg deficiency undermining immune tolerance (172). Murine lupus models likewise demonstrated the ability of rapamycin to normalize lymphocyte function, the treated animals having significantly reduced autoantibody levels and reduced activation markers on T and B cell compartments (173). These combined observations place mTOR inhibition as a means to reverse senescence-induced immune dysregulation. Combination therapy using rapamycin and modified IL-2 constructs demonstrates synergistic effects in autoimmune conditions, particularly by reinforcing Treg cell function. In models of T1D and primary biliary cholangitis, this dual approach markedly improved Treg survival and functional stability—effectively counteracting the progressive regulatory decline characteristic of aging immune systems (112). Therapeutic application of rapamycin and rapalogues is also broadened to other autoimmune conditions with chronic immune activation and accelerated immune aging. Therapeutic benefits have been observed in Sjögren syndrome (118), juvenile idiopathic arthritis (JIA) (117) and RA (114), wherein not only are the overactive immune responses suppressed but also the pathologies of senescence below them can be addressed. Most notably in RA, rapamycin targets pathologic aging of fibroblast-like synoviocytes and autoreactive lymphocytes- the key cellular propagators of irreversible joint damage (174, 175).

The simultaneous modulation of immune and senescence pathways by rapamycin is a unique therapeutic strategy—selective reprogramming rather than elimination of senescent immune cells. This dual mechanism has the potential to mediate autoimmunity with the immunological triad of age-related disease and inflammation and cellular senescence. As outlined in **Table 1**, preclinical models position rapamycin as an autoimmune model for immune cell senescence therapies.

##### 4.1.2.2 Metformin

Beyond its established role in glucose regulation, metformin also exerts significant immunomodulatory effects that are able to influence cellular aging pathways (119). The primary action of the drug is the activation of AMP-activated protein kinase (AMPK) with subsequent inhibition of mTOR signaling—an action central to senescence regulation and suppression of inflammatory mediators (176). Through such metabolic crosstalk, metformin seems to counteract the pro-inflammatory milieu induced by senescent subsets of immune cells in MS (177), RA (178), and SLE (179), as reported in **Table 1**.

Several lines of evidence suggest that metformin reverses aging T cell function via metabolic reprogramming. The three senescence-associated defects that the drug acts on are diminished mitochondrial effectiveness, hyperproduction of ROS, and deregulated NF-κB signaling—all reducing pro-inflammatory secretory profiles (180). In EAE models, metformin shows neuroprotection, with reduced CNS demyelination, regulated microglial function, and less invasion by senescent immune cells (121). Besides, metformin possesses microbiome-modulating activity that can play a role in immune regulation. The medication restores microbial diversity—across the board perturbed in autoimmunity and aging—with potential to curb gut-derived initiators of systemic inflammation that drive immune aging (181).

##### 4.1.2.3 Resveratrol

The bioactive polyphenol resveratrol (present in many plant species) has antioxidant and immunomodulatory effects that could be potentially therapeutic for autoimmune and age-related immune diseases (**Table 1**) (182). Mechanistically, it interferes with NF-κB activation pathways by blocking IL-1 signaling pathways, thereby impairing the formation of senescence-associated inflammatory mediators. In addition, resveratrol was found to favorably modulate immune polarization by inhibiting pro-inflammatory Th17 reactions while enhancing immunoregulatory T cell populations—remedying an underlying imbalance common to both immune aging and autoimmune disease pathogenesis (183). Experimental investigations reveal resveratrol’s therapeutic potential across multiple autoimmune models. In RA studies using C57BL/6 mice, treatment produced three significant improvements: decreased articular inflammation, reduced nociceptive responses, and suppression of neutrophil extracellular trap generation—all critical factors in autoimmune-mediated joint destruction (184). Demyelination studies using cuprizone-induced animal models demonstrate resveratrol’s neurorestorative capacity, with treatment enhancing oligodendrocyte-mediated myelin regeneration. These findings suggest therapeutic potential for MS by addressing the core pathological feature of axonal insulation loss (185). Collectively, these observations position resveratrol as a promising candidate for reducing aging-related immune dysfunction and its inflammatory consequences. Despite its therapeutic potential, clinical application faces significant pharmacological challenges—including limited aqueous solubility, extensive first-pass metabolism, and less than optimal systemic absorption—that currently limit its translational application (122). Innovative drug delivery approaches overcome the pharmacological limitations of resveratrol. For example, encapsulation of β-lactoglobulin nanoparticles enhances both solubility (200% increment) and therapeutic potency, such that treated models show enhanced production of IL-10—a biomarker of successful immune reprogramming (186). Advanced delivery systems using chitosan nanocomposites enable site-specific release of resveratrol in the colon, demonstrating particular promise for IBD management (123). This targeted approach simultaneously addresses two pathological drivers: SASP activity and T cell imbalance. Resveratrol’s multifaceted immunomodulatory properties establish it as a prototype for plant-derived therapeutics targeting immune cell aging in autoimmunity.

##### 4.1.2.4 Aspirin

The nonsteroidal anti-inflammatory drug (NSAID) aspirin exerts its pharmacological effects by selectively inhibiting the cyclooxygenase (COX) enzyme, a key regulator of thromboxane and inflammatory prostaglandin production. This mechanism underlies both its anti-inflammatory properties and cardiovascular benefits (187). Although aspirin remains a historically important therapy for RA symptom management (124), its clinical use has declined due to adverse gastrointestinal and cardiovascular effects, with newer COX-2 selective agents now favored. However, preclinical research reveals unexpected immunomodulatory properties, positioning aspirin as a potential modifier of immune aging processes (**Table 1**). EAE studies demonstrate aspirin’s capacity to redirect CD4+ T cell differentiation—suppressing the development of pathogenic Th1/Th17 cells while enhancing FOXP3+ Treg cell generation through CREB-dependent IL-11 production (125). Ongoing clinical investigations continue to uncover aspirin’s secondary benefits for autoimmune patients. Retrospective data indicate prophylactic low-dose administration may reduce cardiovascular risk in SLE populations (126)—particularly relevant given their chronic inflammation and accelerated vascular disease progression, both exacerbated by senescence-associated immune dysfunction. These observations highlight aspirin’s potential as a multipurpose therapeutic when judiciously implemented in immunocompromised populations.

##### 4.1.2.5 P38 MAP kinase inhibitors

Autoimmune inflammation is also tightly controlled by certain signaling molecules, among which IL-1 and IL-6 are of special interest. Both of these cytokines are regulated by certain enzymes referred to as MAPKs, which transmit extracellular stress signals to intracellular function. The p38 MAPK pathway has been found to be particularly dysregulated in autoimmune diseases such as RA (188) and IBD (189). After overactivation, it results in the release of inflammatory mediators from senescent cells, becoming involved in a vicious cycle of chronic inflammation that injures the tissues during autoimmunity and regular aging.

Experimental research using the p38 MAPK inhibitor SB203580 has revealed significant disease-modifying effects. In murine models of spontaneous lupus (MRL/lpr strain), oral treatment with this inhibitor produced measurable renal benefits, including decreased urinary protein excretion and structural preservation of renal tissue (128). SB203580 exhibited neuroprotection in models of EAE, retarding disease progression by two mechanisms: suppression of myelin loss by Th17-mediated mechanisms and disruption of ROS accumulation—both of which are central drivers of immune-associated neural degeneration (127). The same pathway may also underlie pemphigus vulgaris epidermal blistering, where the pathogenic autoantibodies induce p38-mediated tissue damage (190). Further studies point to the immunoregulatory receptor Tim-3 regulating p38 signaling to mediate autoimmune hepatitis development in animal models via the inhibition of pathological Th17 activity (130). These findings together emphasize p38 MAPK’s bipotential role in acute autoimmune as well as chronic senescent immune cell-mediated inflammation. Pharmacological inhibition of p38 MAPK provides a dual-action therapeutic strategy to mitigate the SASP and target central autoimmune mechanisms, as summarized in **Table 1**. This dual-action potential makes p38 inhibitors very promising for the therapeutic treatment of age-aggravated autoimmune diseases.

##### 4.1.2.6 JAK/STAT inhibitors

The Janus kinase (JAK)/STAT signaling axis functions as a principal orchestrator of immune activation, with pathway dysregulation now recognized as a fundamental driver of autoimmune pathology (191). Chronic JAK/STAT activation maintains the pathological secretory behavior of senescent immune cells, creating self-perpetuating inflammatory cycles that drive tissue damage (192). Pharmacological interruption of this pathway demonstrates therapeutic potential, as shown by ruxolitinib’s capacity to reduce pathogenic Th17 populations and suppress inflammatory mediators in EAE models, resulting in measurable clinical improvement (193). Animal studies of cutaneous lupus erythematosus (CLE) demonstrate ruxolitinib’s capacity to suppress disease-promoting cytokine networks, suggesting an ability to interrupt the inflammatory feedback cycles that sustain autoimmune tissue damage (131). This preclinical evidence translates to clinical benefit, with topical ruxolitinib formulations showing measurable therapeutic effects in CLE patients (**Table 1**) (194). Psoriasis pathogenesis involves the accumulation of senescent T cell and chronic inflammation, both of which respond to JAK pathway inhibition. Clinical studies reveal significant improvement in psoriatic symptoms with both ruxolitinib (targeting JAK1/2) and tofacitinib (JAK1/3 selective), demonstrating reduced cutaneous inflammation and visible lesion clearance (**Supplementary Table 1**) (132). The JAK inhibitor tofacitinib, formulated for both systemic and localized delivery, has demonstrated clinical efficacy in refractory RA across phase III trials, including patients with inadequate response to conventional therapies such as methotrexate or biologic agents (**Supplementary Table 1**) (132, 195). This class of therapeutics provides combined advantages for autoimmune management by simultaneously disrupting pro-inflammatory signaling cascades and potentially counteracting the pathological effects of senescent immune populations that sustain disease activity.

### 4.2 Immunotherapy approaches

The treatment of autoimmune diseases calls for novel therapeutic strategies in light of their multifactorial etiology. Immunotherapeutic agents have emerged as central tools with their ability to modulate immune activity in a specific manner by various mechanisms. The present review covers the existing immunomodulatory modalities, including antibody-targeted biologics, checkpoint regulators, and adoptive cell therapies. **Supplementary Table 1** highlights notable clinical trial findings that demonstrate the therapeutic effectiveness of these modalities for the treatment of autoimmune diseases.

#### 4.2.1 Monoclonal antibodies

Engineered antibody therapies have also been developed as targeted medicine for autoimmune disease with the potential for selective manipulation of pathogenic immune populations that have accumulated during immune aging. These biologics achieve targeted immune modulation by two main mechanisms: direct cell clone depletion of autoreactive cells and functional reprogramming of senescent lymphocytes. This double feature improves treatment specificity with the possibility to leave behind protective immunity (196). The B-cell activating factor (BAFF)-specific monoclonal antibody belimumab illustrates this therapeutic principle in SLE. By competitively blocking BAFF receptor interactions (TACI, BR3, and BCMA), it disrupts critical survival signals for autoreactive B cell populations - including those developing senescence markers. This targeted intervention selectively removes pathogenic B cell clones while maintaining normal humoral immunity, effectively reducing autoimmune pathology (197–200). B-cell targeting antibodies like rituximab demonstrate broad therapeutic effects through CD20-mediated depletion of pathogenic B lymphocyte populations. This includes elimination of dysfunctional B cells displaying SASP that contribute to inflammatory pathology in SLE (201). Rituximab therapy achieves rapid peripheral B-cell depletion in RA patients, with ≥95% reduction in CD20+ lymphocytes and concomitant decreases in CRP/ESR levels detectable by day 14 post-infusion (202). The agent demonstrates comparable lympholytic efficacy in systemic vasculitides, with phase III trials documenting sustained CD19+ cell counts <5 cells/μL in 89% of granulomatosis with polyangiitis and microscopic polyangiitis patients through 6-month follow-up (203). The therapeutic depletion of B lymphocytes holds particular significance in cellular senescence, given the pathological accumulation of senescent B-cell populations in autoimmune disorders. These dysfunctional cells perpetuate chronic inflammatory states through two principal mechanisms: sustained secretion of pro-inflammatory cytokines, and progressive erosion of immune tolerance pathways (204). The evolution of anti-CD20 biologics has yielded engineered monoclonal antibodies (obinutuzumab, veltuzumab, ofatumumab, ublituximab) demonstrating three key advancements over predecessor molecules: optimized effector function through Fc domain modifications, reduced neutralizing antibody formation via humanized frameworks, and prolonged *in vivo* persistence. Veltuzumab exemplifies this progress—while maintaining rituximab’s target specificity, its humanized variable regions confer both extended circulation time (t½ increased 2.3-fold in clinical studies) and decreased human anti-chimeric antibody (HACA) responses, significantly improving its safety profile (205). Emerging clinical evidence also positions corelizumab as a potent anti-CD20 intervention for MS, demonstrating significant reductions in both relapse frequency (43.7% vs placebo, p<0.001) and disability progression (34% reduction in 12-week CDP) in phase III trials (206). This therapeutic effect appears mediated through selective clearance of senescent CD27+ memory B-cell subsets known to drive neuroinflammation via IL-6 and GM-CSF secretion (207, 208). The mechanistic link between B-cell senescence and autoimmunity further validates anti-CD20 biologics as precision tools for cellular senescence modulation. Ofatumumab exemplifies this approach in RA, where subcutaneous administration achieved ACR50 responses in 41.2% of TNF-α refractory patients (207) and reduced synovial ectopic lymphoid structures by 68% (208), suggesting direct targeting of pathogenic B-cell niches.

Ofatumumab exhibits unique CD20 binding characteristics compared to rituximab, engaging both the small (residues 72-82) and large (residues 142-182) extracellular loops of the target protein through its distinct epitope recognition. This bivalent interaction induces enhanced membrane-bound complement component C1q recruitment, achieving 8-fold greater complement-dependent cytotoxicity *in vitro* (p<0.01). Furthermore, the antibody’s sustained membrane proximity facilitates direct B-cell lysis through lipid raft destabilization, independently of FcγR-mediated effector functions (209). Senescent B cells were revealed to play a role in autoimmune diseases by inducing immune dysregulation through pro-inflammatory chronic signaling. Thus, based on their pathogenic function, treatments, such as ofatumumab, have great promise for reversing cellular senescence effects. Obinutuzumab, another anti-CD20 monoclonal antibody, has been revealed to be effective in clinical manifestations in SLE, the most notable effect being observed in lupus nephritis. Clinical studies show that obinutuzumab not only decreases the risk of worsening kidney function but also has better complete renal response rates compared to placebo (210). These observations indicate obinutuzumab greatly decreases autoreactive and conceivably senescent B cells, which play a central role in initiating chronic inflammation in SLE. At the same time, ublituximab, a monoclonal antibody originally developed to treat chronic lymphocytic leukemia, is being repurposed for relapsing MS, a disease also characterized by immune aging defects. In ULTIMATE I and II phase III trials, ublituximab significantly decreased annualized relapse rates and MRI lesion activity compared to teriflunomide in patients with relapsing MS (211). These findings suggest that ublituximab exerts its therapeutic effects, at least in part, through modulation of immune senescence pathways involved in MS pathogenesis.

CD22 has emerged as an additional therapeutic target for B-cell mediated disease. The humanized anti-CD22 monoclonal antibody epratuzumab has been shown to cause clinically relevant reductions in B-lymphocyte levels in the circulation and titers of IgM, while preserving intact T-cell levels and other immunoglobulin isotypes (212). This pattern of depletion is of particular interest because CD22 functions as a gatekeeper of B-cell receptor signaling thresholds. Such bispecific immunotherapy can provide such new benefits in treating age-related B-cell hyperreactivity in autoimmune diseases. More importantly, new forms of bispecific antibodies against both CD22 and CD20 epitopes (with the help of epratuzumab and veltuzumab derivatives) were also found to be more effective in the inhibition of membrane proteins that are important for B-cell activation and chemotaxis. Such combination therapy is more potentially therapeutic with dual antigen targeting (213).

Emerging immunomodulatory therapies have identified the BLyS-APRIL cytokine network as a key regulator of pathologic B-cell activity in autoimmune disease. The dual antagonist recombinant fusion protein atacicept exhibits a singular efficacy in diseases with chronic autoreactive B-cells. Atacicept-treated lupus nephritis and IgA nephropathy clinical trials showed dramatic reduction in disease biomarkers upon atacicept treatment, paralleled by suppressed inflammatory responses mediated by long-lived B-cell subsets. The therapeutic benefits of this approach have been validated in multiple clinical settings. Research has confirmed significant clinical improvement in autoimmune disorders characterized by pathogenic B-cell accumulation, including active lupus nephritis (214) and IgA nephropathy (215). By modulating these cell populations, atacicept offers a promising avenue for restoring immune balance in aging-related autoimmunity. Another immunotherapeutic strategy targeted the CD11a subunit of LFA-1 through the monoclonal antibody efalizumab, originally developed for psoriatic disease. Efalizumab functioned by blocking T-cell activation and cutaneous trafficking, effectively reducing dermal infiltration and keratinocyte hyperproliferation—hallmarks of psoriatic plaques. While early clinical results were promising, post-marketing safety concerns led to its withdrawal from the global market (216). Future treatments can aim the effector T cells in such a manner so that they do not cause chronic inflammation caused by senescent immune cells as a strategy. These cells can cause SASP, a causative factor of pathology by releasing high levels of inflammatory mediators like TNF-α and IL-6. The chronic inflammatory tissue environment triggers additional ongoing tissue damage and loss of immune homeostasis. Current studies indicate that selective inhibition of SASP constituents would be a suitable therapy to correct autoimmune function in aging-related autoimmune diseases and offers new therapeutic possibilities. TNF-α inhibitors, including etanercept and adalimumab, have proven effective in a wide range of autoimmune diseases—psoriatic arthritis (217, 218), psoriasis (219), RA (218, 220), ulcerative colitis (221, 222), Crohn’s disease, and ankylosing spondylitis (218, 223). The therapeutic benefits of these agents may stem, at least partially, from their ability to counteract senescence-driven inflammation, thereby restoring immune equilibrium and mitigating tissue injury. This mechanism aligns with observations from clinical studies of tocilizumab, an IL-6 receptor blocker, which has shown measurable efficacy in autoimmune disorders where inflammation is exacerbated by immunosenescent processes. These include refractory polymyositis and dermatomyositis (224), giant cell arteritis (225), relapsing polychondritis (226), primary Sjögren’s syndrome (227), Graves’ ophthalmopathy (228), and hemophagocytic lymphohistiocytosis (229). Tocilizumab induces its therapeutic activity through inhibition of IL-6 signaling, thus resolving SASP-mediated inflammation and inducing immune homeostasis. Analogously, the proteasome inhibitor bortezomib has been considered for the therapy of autoimmune conditions with immune cell senescence. Its use for inapproapriate immune response modulation is most applicable in conditions of disease when autoreactive plasma cells are accumulated as a result of age-associated immune deregulation, e.g., refractory warm autoimmune hemolytic anemia. In addition, combination therapy with agents such as cyclophosphamide or rituximab potentiates bortezomib’s effect by increasing immunosuppression and reducing pathogenic levels of B cells (230). There is increasingly available evidence to suggest the application of multi-drug regimens for evading the compromised pathways of cell survival seen in aged immune status. As a critical regulator of inflammatory signaling, the JAK-linked kinase TYK2 generally possesses aberrant activity in immunosenescent states. Such dysregulation is crucially implicated in autoimmune pathogenesis, especially in chronic inflammatory diseases such as psoriasis where immune aging is a critical component. Deucravacitinib’s selective TYK2 inhibition is an important therapeutic innovation. Clinical data in rigorous Phase II/III clinicals in plaque psoriasis patients were adequate efficacy and safety to seek FDA approval. Its mechanism of action is also well suited to inhibit autoimmune activity while restoring some remaining residual impaired age-related immune activity (231). Additional research is left to determine these biotherapeutic agents, and complete trial results are presented in **Supplementary Table 1**.

#### 4.2.2 CAR-T cell therapy targeting senescent cells

Recent developments in autoimmune disease therapy have identified two potentially profitable immunomodulatory strategies: chimeric antigen receptor (CAR) T-cell and monoclonal antibody therapies. Although monoclonal antibodies initially were effective against hematologic malignancies and possess the dominant mechanism by peripheral B-cell depletion, CAR-T therapy is a targeted precision medicine strategy involving the genetic manipulation of autologous T cells to recognize and destroy harmful immune cell populations. One of the more promising features of CAR-T cells is their known capacity for invading immunologically privileged areas, such as the CNS—therapeutic effect not typically observed with standard B-cell depleting therapies.

The treatment setting for autoimmune disorders also encompasses several CD19-targeting CAR-T cell strategies in late-stage clinical evaluation. One of such drugs, CC-97540, is being evaluated in lupus patients via an open-label multicenter trial with an active phase I (NCT05869955) (232). Another strategy, KYV-101, incorporates fully humanized anti-CD19 CAR-T cells and is undergoing phase I/II development for challenging-to-treat lupus nephritis (NCT05938725) (233). Regulatory milestones have included recent FDA clearance for starting in phase II testing of treatment-refractory progressive MS (NCT06384976) (234). Additional trials are exploring CAR-T therapy in other autoimmune indications, including stiff person syndrome (NCT06588491) (235) and generalized myasthenia gravis (NCT06193889) (236). The treatment effectiveness of CAR-T cell therapies can be compromised by cellular senescence. Aged T cells exhibit biased proliferative capacity downregulation, increased markers of exhaustion such as PD-1 and LAG-3, and decreased longevity following adoptive transfer—characteristics singly or cumulatively decreasing therapeutic effectiveness. This limitation is additionally compounded by the pro-inflammatory environment created by frequent SASP factor secretion from senescent immune cells, which could compromise patients to poorly intended inflammatory complications like the cataclysmic cytokine release syndrome. Despite these challenges, next-generation CAR-T designs are incorporating advanced engineering solutions. Neutralizing these limitations, third-generation CAR-T platforms now include advanced engineering strategies for overcoming immunosenescent challenges. Engineered co-stimulation domains—namely, CD28 and 4-1BB mutants—have been engineered with strategic design to augment T cell activation and induce cellular duration of action in senescent immune environments. At the same time, researchers are utilizing gene-editing technologies to overcome built-in inhibitory strategies through modulation of key checkpoint molecules such as PD-1 and CTLA-4 (237). Murine models have shown that urokinase-type plasminogen activator receptor (uPAR)-targeting CAR-T cells are capable of effectively eliminating senescent cells in intestinal tissue. They not only eliminated the old cellular populations with efficacy but also enabled two important secondary effects: efficient downregulation of MHC class II molecules on epithelial surfaces and restoration of gut mucosal barrier function. These observations highlight the two-pronged therapeutic value of this method, with the ability to cure cellular senescence as well as its resultant immunological effects in aging diseases (238, 239). These findings collectively underscore the dual potential of CAR-T cell therapy: not only in targeting autoreactive immune cells, but also in mitigating the detrimental effects of cellular senescence.

## 5 Concluding remarks

Aging progressively weakens the immune system, fostering susceptibility to autoimmune diseases. This review has examined the role of immune cell senescence in autoimmunity pathogenesis—both as a driver of the disease and as a target for treatment. Cellular aging progressively impairs immune cells’ capacity to maintain self-tolerance, inducing chronic inflammation by means of the SASP and compromised immune surveillance. The pathophysiology underlying lies in complex interactions between senescent T cells, B cells, and innate immune loops, which all work together to maintain a chronic inflammatory environment that conditions autoimmune phenotypes. Energetic therapies such as senolytics, xenomorphics, and second-generation immunotherapies hold the potential for reversal of cellular senescence. These therapies not only reverse immune function but also provide opportunities for age-related autoimmunity to be treated with individualized therapy regimens. Current research involves the combination of anti-senescence therapy with traditional therapy to attain optimum effectiveness with fewer adverse effects. Enhanced knowledge on immune cell senescence will form the core of subsequent advances in therapeutics that enhances immune homeostasis and quality of life in individuals affected by autoimmune diseases.

## 6 Future directions

The expanding armory of targeted therapeutics against autoimmune diseases is a reflection of the unprecedented advances that have been achieved. As knowledge about immune cells aging unfolds, the strategies outlined here form a sound foundation on which to construct future treatment development. New approaches will probably be an extension of ongoing clinical trials, optimizing current therapy, optimizing patient-individualized therapy, and adding new modalities—e.g., combination therapy or novel technologies—to overcome current therapeutic constraints. Future research will need to concentrate on bridging the gap between fundamental findings and their clinical utility, where mechanistic information is appropriately translated into better outcomes and patient care.

## References

[1] Goodnow CC. Multistep pathogenesis of autoimmune disease. Cell. (2007) 130:25–35. doi: 10.1016/j.cell.2007.06.033, PMID: 17632054

[2] Raphael I Nalawade S Eagar TN Forsthuber TG. T cell subsets and their signature cytokines in autoimmune and inflammatory diseases. Cytokine. (2015) 74:5–17. doi: 10.1016/j.cyto.2014.09.011, PMID: 25458968 PMC4416069

[3] Wahren-Herlenius M Dörner T. Immunopathogenic mechanisms of systemic autoimmune disease. Lancet. (2013) 382:819–31. doi: 10.1016/S0140-6736(13)60954-X, PMID: 23993191

[4] Cho JH Gregersen PK. Genomics and the multifactorial nature of human autoimmune disease. New Engl J Med. (2011) 365:1612–23. doi: 10.1056/NEJMra1100030, PMID: 22029983

[5] Kim N-H Sim S-J Han H-G Yoon J-H Han Y-H. Immunosenescence and age-related immune cells: causes of age-related diseases. Arch Pharmacal Res. (2024) 48:132–49. doi: 10.1007/s12272-024-01529-7, PMID: 39725853

[6] Zhou D Borsa M Simon AK. Hallmarks and detection techniques of cellular senescence and cellular ageing in immune cells. Aging Cell. (2021) 20(2):e13316. 33524238 10.1111/acel.13316 PMC7884036

[7] Wing K Sakaguchi S. Regulatory T cells exert checks and balances on self tolerance and autoimmunity. Nat Immunol. (2010) 11:7–13. doi: 10.1038/ni.1818, PMID: 20016504

[8] Yousefzadeh MJ Flores RR Zhu Y Schmiechen ZC Brooks RW Trussoni CE. An aged immune system drives senescence and ageing of solid organs. Nature. (2021) 594:100–5. doi: 10.1038/s41586-021-03547-7, PMID: 33981041 PMC8684299

[9] Di Micco R Krizhanovsky V Baker D d’Adda di Fagagna F. Cellular senescence in ageing: from mechanisms to therapeutic opportunities. Nat Rev Mol Cell Biol. (2021) 22:75–95. doi: 10.1038/s41580-020-00314-w, PMID: 33328614 PMC8344376

[10] Fülöp T Dupuis G Witkowski JM Larbi A. The role of immunosenescence in the development of age-related diseases. Rev investigacion clinica. (2016) 68:84–91. doi: 10.1016/S0034-8376(25)00212-8, PMID: 27103044

[11] Mittelbrunn M Kroemer G. Hallmarks of T cell aging. Nat Immunol. (2021) 22:687–98. doi: 10.1038/s41590-021-00927-z, PMID: 33986548

[12] Liu X Hoft DF Peng G. Senescent T cells within suppressive tumor microenvironments: emerging target for tumor immunotherapy. J Clin Invest. (2020) 130:1073–83. doi: 10.1172/JCI133679, PMID: 32118585 PMC7269563

[13] Goronzy JJ Weyand CM. Ageing, autoimmunity and arthritis: T-cell senescence and contraction of T-cell repertoire diversity–catalysts of autoimmunity and chronic inflammation. Arthritis Res Ther. (2003) 5:225–34. doi: 10.1186/ar974, PMID: 12932282 PMC193735

[14] Johnson SA Cambier JC. Ageing, autoimmunity and arthritis: senescence of the B cell compartment–implications for humoral immunity. Arthritis Res Ther. (2004) 6:1–9. doi: 10.1186/ar1180, PMID: 15225355 PMC464870

[15] Castro F Cardoso AP Gonçalves RM Serre K Oliveira MJ. Interferon-gamma at the crossroads of tumor immune surveillance or evasion. Front Immunol. (2018) 9:847. doi: 10.3389/fimmu.2018.00847, PMID: 29780381 PMC5945880

[16] Zhang J He T Xue L Guo H. Senescent T cells: a potential biomarker and target for cancer therapy. EBioMedicine. (2021) 68:103409. doi: 10.1016/j.ebiom.2021.103409, PMID: 34049248 PMC8170103

[17] Lanna A Henson SM Escors D Akbar AN. The kinase p38 activated by the metabolic regulator AMPK and scaffold TAB1 drives the senescence of human T cells. Nat Immunol. (2014) 15:965–72. doi: 10.1038/ni.2981, PMID: 25151490 PMC4190666

[18] Lanna A Coutavas E Levati L Seidel J Rustin MH Henson SM. IFN-α inhibits telomerase in human CD8+ T cells by both hTERT downregulation and induction of p38 MAPK signaling. J Immunol. (2013) 191:3744–52. doi: 10.4049/jimmunol.1301409, PMID: 23997212 PMC3836248

[19] Lanna A Gomes DC Muller-Durovic B McDonnell T Escors D Gilroy DW. A sestrin-dependent Erk–Jnk–p38 MAPK activation complex inhibits immunity during aging. Nat Immunol. (2017) 18:354–63. doi: 10.1038/ni.3665, PMID: 28114291 PMC5321575

[20] Strioga M Pasukoniene V Characiejus D. CD8+ CD28– and CD8+ CD57+ T cells and their role in health and disease. Immunology. (2011) 134:17–32. doi: 10.1111/j.1365-2567.2011.03470.x, PMID: 21711350 PMC3173691

[21] Chattopadhyay PK Betts MR Price DA Gostick E Horton H Roederer M. The cytolytic enzymes granyzme A, granzyme B, and perforin: expression patterns, cell distribution, and their relationship to cell maturity and bright CD57 expression. J Leucocyte Biol. (2009) 85:88–97. doi: 10.1189/jlb.0208107, PMID: 18820174 PMC2638730

[22] Henson SM. CD8+ T-cell senescence: No role for mTOR. Biochem Soc Trans. (2015) 43:734–9. doi: 10.1042/BST20150092, PMID: 26551721

[23] Yang M Zhu L. Osteoimmunology: the crosstalk between T cells, B cells, and osteoclasts in rheumatoid arthritis. Int J Mol Sci. (2024) 25:2688. doi: 10.3390/ijms25052688, PMID: 38473934 PMC10931770

[24] Karnowski A Chevrier S Belz GT Mount A Emslie D D’Costa K. B and T cells collaborate in antiviral responses via IL-6, IL-21, and transcriptional activator and coactivator, Oct2 and OBF-1. J Exp Med. (2012) 209:2049–64. doi: 10.1084/jem.20111504, PMID: 23045607 PMC3478936

[25] Pawlik A Ostanek L Brzosko I Brzosko M Masiuk M Machalinski B. The expansion of CD4+ CD28-T cells in patients with rheumatoid arthritis. Arthritis Res Ther. (2003) 5:1–4. doi: 10.1186/ar766, PMID: 12823856 PMC165060

[26] Gao Y Cai W Zhou Y Li Y Cheng J Wei F. Immunosenescence of T cells: a key player in rheumatoid arthritis. Inflammation Res. (2022) 71:1449–62. doi: 10.1007/s00011-022-01649-0, PMID: 36280621

[27] Giubilato S Liuzzo G Brugaletta S Pitocco D Graziani F Smaldone C. Expansion of CD4+ CD28null T-lymphocytes in diabetic patients: exploring new pathogenetic mechanisms of increased cardiovascular risk in diabetes mellitus. Eur Heart J. (2011) 32:1214–26. doi: 10.1093/eurheartj/ehq499, PMID: 21217142

[28] Markovic-Plese S Cortese I Wandinger K-P McFarland HF Martin R. CD4+ CD28–costimulation-independent T cells in multiple sclerosis. J Clin Invest. (2001) 108:1185–94. doi: 10.1172/JCI200112516 PMC209525 11602626

[29] Sun Z Zhong W Lu X Shi B Zhu Y Chen L. Association of graves’ Disease and prevalence of circulating IFN-γ-producing CD28– T cells. J Clin Immunol. (2008) 28:464–72. doi: 10.1007/s10875-008-9213-4, PMID: 18704663

[30] Lamprecht P Moosig F Csernok E Seitzer U Schnabel A Mueller A. CD28 negative T cells are enriched in granulomatous lesions of the respiratory tract in Wegener’s granulomatosis. Thorax. (2001) 56:751–7. doi: 10.1136/thorax.56.10.751, PMID: 11562512 PMC1745938

[31] Sawai H Park YW Roberson J Imai T Goronzy JJ Weyand CM. T cell costimulation by fractalkine-expressing synoviocytes in rheumatoid arthritis. Arthritis Rheumatism. (2005) 52:1392–401. doi: 10.1002/art.21140, PMID: 15880821

[32] Broux B Pannemans K Zhang X Markovic-Plese S Broekmans T Eijnde BO. CX3CR1 drives cytotoxic CD4+ CD28– T cells into the brain of multiple sclerosis patients. J autoimmunity. (2012) 38:10–9. doi: 10.1016/j.jaut.2011.11.006, PMID: 22123179

[33] Xu W Larbi A. Markers of T cell senescence in humans. Int J Mol Sci. (2017) 18:1742. doi: 10.3390/ijms18081742, PMID: 28796199 PMC5578132

[34] Dehbi AZ Radstake TR Broen JC. Accelerated telomere shortening in rheumatic diseases: cause or consequence? Expert Rev Clin Immunol. (2013) 9:1193–204. doi: 10.1586/1744666X.2013.850031, PMID: 24215409

[35] Handono K Wahono CS Pratama MZ Kalim H. Association of the premature immunosenescence with the presence and severity of anemia among patients with systemic lupus erythematosus. Lupus. (2021) 30:1906–14. doi: 10.1177/09612033211038057, PMID: 34720016

[36] LeBien TW Tedder TF. B lymphocytes: how they develop and function. Blood J Am Soc Hematol. (2008) 112:1570–80. doi: 10.1182/blood-2008-02-078071, PMID: 18725575 PMC2518873

[37] Claes N Fraussen J Vanheusden M Hellings N Stinissen P Van Wijmeersch B. Age-associated B cells with proinflammatory characteristics are expanded in a proportion of multiple sclerosis patients. J Immunol. (2016) 197:4576–83. doi: 10.4049/jimmunol.1502448, PMID: 27837111

[38] Adlowitz DG Barnard J Biear JN Cistrone C Owen T Wang W. Expansion of activated peripheral blood memory B cells in rheumatoid arthritis, impact of B cell depletion therapy, and biomarkers of response. PloS One. (2015) 10:e0128269. doi: 10.1371/journal.pone.0128269, PMID: 26047509 PMC4457888

[39] de Mol J Kuiper J Tsiantoulas D Foks AC. The dynamics of B cell aging in health and disease. Front Immunol. (2021) 12:733566. doi: 10.3389/fimmu.2021.733566, PMID: 34675924 PMC8524000

[40] Frasca D Blomberg BB. Aging induces B cell defects and decreased antibody responses to influenza infection and vaccination. Immun Ageing. (2020) 17:1–10. doi: 10.1186/s12979-020-00210-z, PMID: 33292323 PMC7674578

[41] Lescale C Dias S Maës J Cumano A Szabo P Charron D. Reduced EBF expression underlies loss of B-cell potential of hematopoietic progenitors with age. Aging Cell. (2010) 9:410–9. doi: 10.1111/j.1474-9726.2010.00566.x, PMID: 20331442

[42] Anspach J Poulsen G Kaattari I Pollock R Zwollo P. Reduction in DNA binding activity of the transcription factor Pax-5a in B lymphocytes of aged mice. J Immunol. (2001) 166:2617–26. doi: 10.4049/jimmunol.166.4.2617, PMID: 11160324

[43] Rodriguez-Zhurbenko N Quach TD Hopkins TJ Rothstein TL Hernandez AM. Human B-1 cells and B-1 cell antibodies change with advancing age. Front Immunol. (2019) 10:483. doi: 10.3389/fimmu.2019.00483, PMID: 30941130 PMC6433875

[44] Almanan M Raynor J Ogunsulire I Malyshkina A Mukherjee S Hummel SA. IL-10–producing Tfh cells accumulate with age and link inflammation with age-related immune suppression. Sci Adv. (2020) 6:eabb0806. doi: 10.1126/sciadv.abb0806, PMID: 32832688 PMC7439492

[45] Frasca D Romero M Diaz A Garcia D Thaller S Blomberg BB. B cells with a senescent-associated secretory phenotype accumulate in the adipose tissue of individuals with obesity. Int J Mol Sci. (2021) 22:1839. doi: 10.3390/ijms22041839, PMID: 33673271 PMC7917792

[46] Frasca D Romero M Garcia D Thaller S Bueno V. Adipocyte-derived inflammatory molecules induce senescent B cells through metabolic pathways. Obesity. (2024) 32(8):1441–7. doi: 10.1002/oby.24013, PMID: 38575197 PMC11269042

[47] Gray M Miles K Salter D Gray D Savill J. Apoptotic cells protect mice from autoimmune inflammation by the induction of regulatory B cells. Proc Natl Acad Sci. (2007) 104:14080–5. doi: 10.1073/pnas.0700326104, PMID: 17715067 PMC1955797

[48] Matsumoto M Baba A Yokota T Nishikawa H Ohkawa Y Kayama H. Interleukin-10-producing plasmablasts exert regulatory function in autoimmune inflammation. Immunity. (2014) 41:1040–51. doi: 10.1016/j.immuni.2014.10.016, PMID: 25484301

[49] Budzynski W Radzikowski C. Cytotoxic Cs in immunodeficient athymic mice. Immunopharmacol immunotoxicol. (1994) 16:319–46. doi: 10.3109/08923979409007097, PMID: 7528237

[50] Solana R Tarazona R Gayoso I Lesur O Dupuis G Fulop T. Innate immunosenescence: effect of aging on cells and receptors of the innate immune system in humans. Semin Immunol. (2012) 24(5):331–41. doi: 10.1016/j.smim.2012.04.008, PMID: 22560929

[51] Toomey JA Gays F Foster D Brooks CG. Cytokine requirements for the growth and development of mouse NK cells *in vitro* . J Leucocyte Biol. (2003) 74:233–42. doi: 10.1189/jlb.0303097, PMID: 12885940

[52] King AM Keating P Prabhu A Blomberg BB Riley RL. NK cells in the CD19– B220+ bone marrow fraction are increased in senescence and reduce E2A and surrogate light chain proteins in B cell precursors. Mech Ageing Dev. (2009) 130:384–92. doi: 10.1016/j.mad.2009.03.002, PMID: 19428458 PMC2743292

[53] Warrington KJ Takemura S Goronzy JJ Weyand CM. CD4+, CD28– T cells in rheumatoid arthritis patients combine features of the innate and adaptive immune systems. Arthritis Rheumatism. (2001) 44:13–20. doi: 10.1002/1529-0131(200101)44:1<13::AID-ANR3>3.0.CO;2-6, PMID: 11212151

[54] Weyand CM Goronzy JJ. Premature immunosenescence in rheumatoid arthritis. J Rheumatol. (2002) 29:1141–6. 12064826

[55] Van Belle TL von Herrath MG. The role of the activating receptor NKG2D in autoimmunity. Mol Immunol. (2009) 47:8–11. doi: 10.1016/j.molimm.2009.02.023, PMID: 19286259

[56] Lu Y Ruan Y Hong P Rui K Liu Q Wang S. T-cell senescence: A crucial player in autoimmune diseases. Clin Immunol. (2023) 248:109202. doi: 10.1016/j.clim.2022.109202, PMID: 36470338

[57] Park YW Kee SJ Cho YN Lee EH Lee HY Kim EM. Impaired differentiation and cytotoxicity of natural killer cells in systemic lupus erythematosus. Arthritis Rheumatism. (2009) 60:1753–63. doi: 10.1002/art.24556, PMID: 19479851

[58] Huang Z Fu B Zheng SG Li X Sun R Tian Z. Involvement of CD226+ NK cells in immunopathogenesis of systemic lupus erythematosus. J Immunol. (2011) 186:3421–31. doi: 10.4049/jimmunol.1000569, PMID: 21296979 PMC3097030

[59] Hodge DL Berthet C Coppola V Kastenmüller W Buschman MD Schaughency PM. IFN-gamma AU-rich element removal promotes chronic IFN-gamma expression and autoimmunity in mice. J autoimmunity. (2014) 53:33–45. doi: 10.1016/j.jaut.2014.02.003, PMID: 24583068 PMC4148478

[60] Liu M Liu J Hao S Wu P Zhang X Xiao Y. Higher activation of the interferon-gamma signaling pathway in systemic lupus erythematosus patients with a high type I IFN score: relation to disease activity. Clin Rheumatol. (2018) 37:2675–84. doi: 10.1007/s10067-018-4138-7, PMID: 29774490

[61] Collin M McGovern N Haniffa M. Human dendritic cell subsets. Immunology. (2013) 140:22–30. doi: 10.1111/imm.12117, PMID: 23621371 PMC3809702

[62] Panda A Qian F Mohanty S Van Duin D Newman FK Zhang L. Age-associated decrease in TLR function in primary human dendritic cells predicts influenza vaccine response. J Immunol. (2010) 184:2518–27. doi: 10.4049/jimmunol.0901022, PMID: 20100933 PMC3867271

[63] Shaw AC Goldstein DR Montgomery RR. Age-dependent dysregulation of innate immunity. Nat Rev Immunol. (2013) 13:875–87. doi: 10.1038/nri3547, PMID: 24157572 PMC4096436

[64] Nyugen J Agrawal S Gollapudi S Gupta S. Impaired functions of peripheral blood monocyte subpopulations in aged humans. J Clin Immunol. (2010) 30:806–13. doi: 10.1007/s10875-010-9448-8, PMID: 20703784 PMC2970801

[65] Seidler S Zimmermann HW Bartneck M Trautwein C Tacke F. Age-dependent alterations of monocyte subsets and monocyte-related chemokine pathways in healthy adults. BMC Immunol. (2010) 11:1–11. doi: 10.1186/1471-2172-11-30, PMID: 20565954 PMC2910032

[66] Yang X Chang Y Wei W. Emerging role of targeting macrophages in rheumatoid arthritis: focus on polarization, metabolism and apoptosis. Cell proliferation. (2020) 53:e12854. doi: 10.1111/cpr.12854, PMID: 32530555 PMC7377929

[67] Debacq-Chainiaux F Erusalimsky JD Campisi J Toussaint O. Protocols to detect senescence-associated beta-galactosidase (SA-βgal) activity, a biomarker of senescent cells in culture and *in vivo* . Nat Protoc. (2009) 4:1798–806. doi: 10.1038/nprot.2009.191, PMID: 20010931

[68] Del Rey MJ Valín Á Usategui A Ergueta S Martín E Municio C. Senescent synovial fibroblasts accumulate prematurely in rheumatoid arthritis tissues and display an enhanced inflammatory phenotype. Immun Ageing. (2019) 16:1–9. doi: 10.1186/s12979-019-0169-4, PMID: 31708994 PMC6833299

[69] Wright HL Moots RJ Edwards SW. The multifactorial role of neutrophils in rheumatoid arthritis. Nat Rev Rheumatol. (2014) 10:593–601. doi: 10.1038/nrrheum.2014.80, PMID: 24914698

[70] Diana J Simoni Y Furio L Beaudoin L Agerberth B Barrat F. Crosstalk between neutrophils, B-1a cells and plasmacytoid dendritic cells initiates autoimmune diabetes. Nat Med. (2013) 19:65–73. doi: 10.1038/nm.3042, PMID: 23242473

[71] Herrero C Marqués L Lloberas J Celada A. IFN-γ–dependent transcription of MHC class II IA is impaired in macrophages from aged mice. J Clin Invest. (2001) 107:485–93. doi: 10.1172/JCI11696, PMID: 11181648 PMC199261

[72] Medzhitov R Horng T. Transcriptional control of the inflammatory response. Nat Rev Immunol. (2009) 9:692–703. doi: 10.1038/nri2634, PMID: 19859064

[73] Metcalf TU Wilkinson PA Cameron MJ Ghneim K Chiang C Wertheimer AM. Human monocyte subsets are transcriptionally and functionally altered in aging in response to pattern recognition receptor agonists. J Immunol. (2017) 199:1405–17. doi: 10.4049/jimmunol.1700148, PMID: 28696254 PMC5548610

[74] Wang X Malawista A Qian F Ramsey C Allore HG Montgomery RR. Age-related changes in expression and signaling of TAM receptor inflammatory regulators in monocytes. Oncotarget. (2018) 9:9572. doi: 10.18632/oncotarget.23851, PMID: 29515754 PMC5839385

[75] Zhang X Dai J Li L Chen H Chai Y. NLRP3 inflammasome expression and signaling in human diabetic wounds and in high glucose induced macrophages. J Diabetes Res. (2017) 2017:5281358. doi: 10.1155/2017/5281358, PMID: 28164132 PMC5259616

[76] Cunha A Perazzio S. Effects of immune exhaustion and senescence of innate immunity in autoimmune disorders. Braz J Med Biol Res. (2024) 57:e13225. doi: 10.1590/1414-431x2024e13225, PMID: 38896644 PMC11186593

[77] Fresneda Alarcon M McLaren Z Wright HL. Neutrophils in the pathogenesis of rheumatoid arthritis and systemic lupus erythematosus: same foe different MO. Front Immunol. (2021) 12:649693. doi: 10.3389/fimmu.2021.649693, PMID: 33746988 PMC7969658

[78] Stoffels K Overbergh L Giulietti A Kasran A Bouillon R Gysemans C. NOD macrophages produce high levels of inflammatory cytokines upon encounter of apoptotic or necrotic cells. J autoimmunity. (2004) 23:9–15. doi: 10.1016/j.jaut.2004.03.012, PMID: 15236748

[79] de Jong T Semmelink J Bolt J Grasso C Hoebe R Krawczyk P. Senolytic treatment to rescue hallmarks of senescence in lymph node fibroblasts from patients with rheumatoid arthritis: Implications for premature aging and potential therapeutic intervention in early rheumatoid arthritis. Clin Exp Immunol. (2025) 219(1):uxaf029. doi: 10.1093/cei/uxaf029, PMID: 40336246 PMC12188290

[80] Drake SS Zaman A Gianfelice C Hua EM-L Heale K Afanasiev E. Senolytic treatment diminishes microglia and decreases severity of experimental autoimmune encephalomyelitis. J Neuroinflammation. (2024) 21:1–18. doi: 10.1186/s12974-024-03278-2, PMID: 39487537 PMC11529445

[81] Jiang J Yang M Zhu H Long D He Z Liu J. CD4+ CD57+ senescent T cells as promoters of systemic lupus erythematosus pathogenesis and the therapeutic potential of senolytic BCL-2 inhibitor. Eur J Immunol. (2024) 57(7):e2350603. doi: 10.1002/eji.202350603, PMID: 38752316

[82] Bardwell PD Gu J McCarthy D Wallace C Bryant S Goess C. The Bcl-2 family antagonist ABT-737 significantly inhibits multiple animal models of autoimmunity. J Immunol. (2009) 182:7482–9. doi: 10.4049/jimmunol.0802813, PMID: 19494271

[83] Jiang J Zhu H Yang M Yang B Wu H Lu Q. Topical administration of a BCL-2 inhibitor alleviates cutaneous lupus erythematosus. Int Immunopharmacol. (2024) 142:113132. doi: 10.1016/j.intimp.2024.113132, PMID: 39288621

[84] Shimp SK Chafin CB Regna NL Hammond SE Read MA Caudell DL. Heat shock protein 90 inhibition by 17-DMAG lessens disease in the MRL/lpr mouse model of systemic lupus erythematosus. Cell Mol Immunol. (2012) 9:255–66. doi: 10.1038/cmi.2012.5, PMID: 22543833 PMC4012849

[85] Hong L-J Chen A-J Li F-Z Chen K-J Fang S. The HSP90 inhibitor, 17-AAG, influences the activation and proliferation of T lymphocytes via AKT/GSK3β signaling in MRL/lpr mice. Drug Design Dev Ther. (2020), 4605–12. doi: 10.2147/DDDT.S269725, PMID: 33149557 PMC7605613

[86] Koga H Prost-Squarcioni C Iwata H Jonkman MF Ludwig RJ Bieber K. Epidermolysis bullosa acquisita: the 2019 update. Front Med. (2019) 5:362. doi: 10.3389/fmed.2018.00362, PMID: 30687710 PMC6335340

[87] Ma C Chen J Li P. Geldanamycin induces apoptosis and inhibits inflammation in fibroblast-like synoviocytes isolated from rheumatoid arthritis patients. J Cell Biochem. (2019) 120:16254–63. doi: 10.1002/jcb.28906, PMID: 31087698

[88] Saeki Y Okita Y Igashira-Oguro E Udagawa C Murata A Tanaka T. Modulation of TNFR 1-triggered two opposing signals for inflammation and apoptosis via RIPK 1 disruption by geldanamycin in rheumatoid arthritis. Clin Rheumatol. (2021) 40:2395–405. doi: 10.1007/s10067-021-05579-w, PMID: 33415454

[89] Tukaj S Sitko K. Heat shock protein 90 (Hsp90) and Hsp70 as potential therapeutic targets in autoimmune skin diseases. Biomolecules. (2022) 12:1153. doi: 10.3390/biom12081153, PMID: 36009046 PMC9405624

[90] Liu Y Ye J Shin Ogawa L Inoue T Huang Q Chu J. The HSP90 inhibitor ganetespib alleviates disease progression and augments intermittent cyclophosphamide therapy in the MRL/lpr mouse model of systemic lupus erythematosus. PloS One. (2015) 10:e0127361. doi: 10.1371/journal.pone.0127361, PMID: 25974040 PMC4431681

[91] Rice JW Veal JM Fadden RP Barabasz AF Partridge JM Barta TE. Small molecule inhibitors of Hsp90 potently affect inflammatory disease pathways and exhibit activity in models of rheumatoid arthritis. Arthritis Rheumatism. (2008) 58:3765–75. doi: 10.1002/art.24047, PMID: 19035474

[92] Zhang L Luo J Wen H Zhang T Zuo X Li X. MDM2 promotes rheumatoid arthritis via activation of MAPK and NF-κB. Int immunopharmacol. (2016) 30:69–73. doi: 10.1016/j.intimp.2015.11.030, PMID: 26655743

[93] Zhang C-x Chen J Cai L Wu J Wang J-y Cao L-f. DNA induction of MDM2 promotes proliferation of human renal mesangial cells and alters peripheral B cells subsets in pediatric systemic lupus erythematosus. Mol Immunol. (2018) 94:166–75. doi: 10.1016/j.molimm.2018.01.003, PMID: 29324237

[94] Ahmed OM Mohamed T Moustafa H Hamdy H Ahmed RR Aboud E. Quercetin and low level laser therapy promote wound healing process in diabetic rats via structural reorganization and modulatory effects on inflammation and oxidative stress. Biomed Pharmacother. (2018) 101:58–73. doi: 10.1016/j.biopha.2018.02.040, PMID: 29477473

[95] Haleagrahara N Miranda-Hernandez S Alim MA Hayes L Bird G Ketheesan N. Therapeutic effect of quercetin in collagen-induced arthritis. Biomed Pharmacother. (2017) 90:38–46. doi: 10.1016/j.biopha.2017.03.026, PMID: 28342364

[96] Kawaguchi K Kaneko M Miyake R Takimoto H Kumazawa Y. Potent inhibitory effects of quercetin on inflammatory responses of collagen-induced arthritis in mice. Endocrine Metab Immune Disorders-Drug Targets (Formerly Curr Drug Targets-Immune Endocrine Metab Disorders). (2019) 19:308–15. doi: 10.2174/1871530319666190206225034, PMID: 30727927

[97] Guazelli CF Staurengo-Ferrari L Zarpelon AC Pinho-Ribeiro FA Ruiz-Miyazawa KW Vicentini FT. Quercetin attenuates zymosan-induced arthritis in mice. Biomed pharmacother. (2018) 102:175–84. doi: 10.1016/j.biopha.2018.03.057, PMID: 29554596

[98] Ju S Ge Y Li P Tian X Wang H Zheng X. Dietary quercetin ameliorates experimental colitis in mouse by remodeling the function of colonic macrophages via a heme oxygenase-1-dependent pathway. Cell Cycle. (2018) 17:53–63. doi: 10.1080/15384101.2017.1387701, PMID: 28976231 PMC5815442

[99] Ijima S Saito Y Nagaoka K Yamamoto S Sato T Miura N. Fisetin reduces the senescent tubular epithelial cell burden and also inhibits proliferative fibroblasts in murine lupus nephritis. Front Immunol. (2022) 13:960601. doi: 10.3389/fimmu.2022.960601, PMID: 36466895 PMC9714549

[100] Guo TL Germolec DR Zheng JF Kooistra L Auttachoat W Smith MJ. Genistein protects female nonobese diabetic mice from developing type 1 diabetes when fed a soy-and alfalfa-free diet. Toxicologic pathol. (2015) 43:435–48. doi: 10.1177/0192623314526318, PMID: 24713318 PMC4190109

[101] You J Cheng J Yu B Duan C Peng J. Baicalin, a Chinese herbal medicine, inhibits the proliferation and migration of human non-small cell lung carcinoma (NSCLC) cells, A549 and H1299, by activating the SIRT1/AMPK signaling pathway. Med Sci monitor: Int Med J Exp Clin Res. (2018) 24:2126. doi: 10.12659/MSM.909627, PMID: 29632297 PMC5909419

[102] Yang X Yang J Zou H. Baicalin inhibits IL-17-mediated joint inflammation in murine adjuvant-induced arthritis. J Immunol Res. (2013) 2013:268065. doi: 10.1155/2013/268065, PMID: 23840239 PMC3694363

[103] Zhong S Ge J Yu JY. Icariin prevents cytokine−induced β−cell death by inhibiting NF−κB signaling. Exp Ther Med. (2018) 16:2756–62. doi: 10.3892/etm.2018.6502, PMID: 30210617 PMC6122516

[104] Gao D heng ZC-c Hao J-p Yang C-c Hu C-y. Icariin ameliorates behavioral deficits and neuropathology in a mouse model of multiple sclerosis. Brain Res. (2023) 1804:148267. doi: 10.1016/j.brainres.2023.148267, PMID: 36731819

[105] Li J Gang D Yu X Hu Y Yue Y Cheng W. Genistein: the potential for efficacy in rheumatoid arthritis. Clin Rheumatol. (2013) 32:535–40. doi: 10.1007/s10067-012-2148-4, PMID: 23307323

[106] Mawarti H Nugraha J Purwanto DA Soeroso J. Environmental factors and protective effects of epigallocatechin-3-gallate to systemic lupus eritematosus: A review study. Indian J Forensic Med Toxicol. (2021) 15:2509–18. doi: 10.37506/ijfmt.v15i2.14750

[107] Jia H Yang Q Hong J Ding R Liu T Yang Q. Microneedles loaded with dexamethasone and epigallocatechin-3-gallate reverse the imbalance of subcutaneous immune homeostasis for the treatment of psoriasis. Advanced Ther. (2024) 7:2300233. doi: 10.1002/adtp.202300233

[108] Abulaban AA Al-Kuraishy HM Al-Gareeb AI Elekhnawy E Alanazi A Alexiou A. Role of fenofibrate in multiple sclerosis. Eur J Med Res. (2024) 29:113. doi: 10.1186/s40001-024-01700-2, PMID: 38336772 PMC10854163

[109] Park A Heo T-H. IL-17A-targeting fenofibrate attenuates inflammation in psoriasis by inducing autophagy. Life Sci. (2023) 326:121755. doi: 10.1016/j.lfs.2023.121755, PMID: 37236601

[110] Calkin A Cooper ME Jandeleit-Dahm K Allen TJ. Gemfibrozil decreases atherosclerosis in experimental diabetes in association with a reduction in oxidative stress and inflammation. Diabetologia. (2006) 49:766–74. doi: 10.1007/s00125-005-0102-6, PMID: 16463048

[111] Lovett-Racke AE Hussain RZ Northrop S Choy J Rocchini A Matthes L. Peroxisome proliferator-activated receptor α agonists as therapy for autoimmune disease. J Immunol. (2004) 172:5790–8. doi: 10.4049/jimmunol.172.9.5790, PMID: 15100326

[112] Kishimoto TK Fournier M Michaud A Rizzo G Roy C Capela T. Rapamycin nanoparticles increase the therapeutic window of engineered interleukin-2 and drive expansion of antigen-specific regulatory T cells for protection against autoimmune disease. J Autoimmunity. (2023) 140:103125. doi: 10.1016/j.jaut.2023.103125, PMID: 37844543

[113] Monti P Scirpoli M Maffi P Piemonti L Secchi A Bonifacio E. Rapamycin monotherapy in patients with type 1 diabetes modifies CD4+ CD25+ FOXP3+ regulatory T-cells. Diabetes. (2008) 57:2341–7. doi: 10.2337/db08-0138, PMID: 18559659 PMC2518485

[114] Bruyn GA Tate G Caeiro F Maldonado-Cocco J Westhovens R Tannenbaum H. Everolimus in patients with rheumatoid arthritis receiving concomitant methotrexate: a 3-month, double-blind, randomised, placebo-controlled, parallel-group, proof-of-concept study. Ann rheumatic diseases. (2008) 67:1090–5. doi: 10.1136/ard.2007.078808, PMID: 18037627

[115] Zhang F Cheng T Zhang S-X. Mechanistic target of rapamycin (mTOR): a potential new therapeutic target for rheumatoid arthritis. Arthritis Res Ther. (2023) 25:187. doi: 10.1186/s13075-023-03181-w, PMID: 37784141 PMC10544394

[116] Song X Gao J Liu H Liu X Tang K. Rapamycin alleviates renal damage in mice with systemic lupus erythematosus through improving immune response and function. Biomed Pharmacother. (2021) 137:111289. doi: 10.1016/j.biopha.2021.111289, PMID: 33581650

[117] Foroncewicz B Mucha K Pączek L Chmura A Rowiński W. Efficacy of rapamycin in patient with juvenile rheumatoid arthritis. Transplant Int. (2005) 18:366–8. doi: 10.1111/j.1432-2277.2004.00070.x, PMID: 15730500

[118] Shah M Edman MC Janga SR Shi P Dhandhukia J Liu S. A rapamycin-binding protein polymer nanoparticle shows potent therapeutic activity in suppressing autoimmune dacryoadenitis in a mouse model of Sjögren’s syndrome. J Controlled release. (2013) 171:269–79. doi: 10.1016/j.jconrel.2013.07.016, PMID: 23892265 PMC3796004

[119] Lin H Ao H Guo G Liu M. The role and mechanism of metformin in inflammatory diseases. J Inflammation Res. (2023) 16:5545–64. doi: 10.2147/JIR.S436147, PMID: 38026260 PMC10680465

[120] Kim DK Park JY Kang YJ Khang D. Drug repositioning of metformin encapsulated in PLGA combined with photothermal therapy ameliorates rheumatoid arthritis. Int J Nanomed. (2023) 18:7267–85. doi: 10.2147/IJN.S438388, PMID: 38090362 PMC10711299

[121] Gilbert EA Livingston J Flores EG Khan M Kandavel H Morshead CM. Metformin treatment reduces inflammation, dysmyelination and disease severity in a mouse model of multiple sclerosis, experimental autoimmune encephalomyelitis. Brain Res. (2024) 1822:148648. doi: 10.1016/j.brainres.2023.148648, PMID: 37890574

[122] Sardou HS Vosough PR Abbaspour M Akhgari A Kesharwani P Sahebkar A. Colon delivery of resveratrol for the treatment of inflammatory bowel disease. J Drug Delivery Sci Technol. (2023) 92:105315. doi: 10.1016/j.jddst.2023.105315 36757584

[123] Iglesias N Galbis E Díaz-Blanco MJ Lucas R Benito E de-Paz M-V. Nanostructured chitosan-based biomaterials for sustained and colon-specific resveratrol release. Int J Mol Sci. (2019) 20:398. doi: 10.3390/ijms20020398, PMID: 30669264 PMC6359380

[124] Fasano S Iacono D Riccardi A Ciccia F Valentini G. The role of aspirin in the primary prevention of accelerated atherosclerosis in systemic autoimmune rheumatic diseases. Rheumatology. (2020) 59:3593–602. doi: 10.1093/rheumatology/keaa335, PMID: 32830272

[125] Mondal S Jana M Dasarathi S Roy A Pahan K. Aspirin ameliorates experimental autoimmune encephalomyelitis through interleukin-11–mediated protection of regulatory T cells. Sci Signaling. (2018) 11:eaar8278. doi: 10.1126/scisignal.aar8278, PMID: 30482850 PMC6325078

[126] Iudici M Fasano S Gabriele Falcone L Pantano I La Montagna G Migliaresi S. Low-dose aspirin as primary prophylaxis for cardiovascular events in systemic lupus erythematosus: a long-term retrospective cohort study. Rheumatology. (2016) 55:1623–30. doi: 10.1093/rheumatology/kew231, PMID: 27247433

[127] Wang L Li B Quan M-Y Li L Chen Y Tan G-J. Mechanism of oxidative stress p38MAPK-SGK1 signaling axis in experimental autoimmune encephalomyelitis (EAE). Oncotarget. (2017) 8:42808. doi: 10.18632/oncotarget.17057, PMID: 28467798 PMC5522107

[128] Jin N Wang Q Zhang X Jiang D Cheng H Zhu K. The selective p38 mitogen-activated protein kinase inhibitor, SB203580, improves renal disease in MRL/lpr mouse model of systemic lupus. Int immunopharmacol. (2011) 11:1319–26. doi: 10.1016/j.intimp.2011.04.015, PMID: 21549858

[129] Nozaki Y Akiba H Akazawa H Yamazawa H Ishimura K Kinoshita K. Inhibition of the TIM-1 and-3 signaling pathway ameliorates disease in a murine model of rheumatoid arthritis. Clin Exp Immunol. (2024) 218:55–64. doi: 10.1093/cei/uxae056, PMID: 38975703 PMC11404125

[130] Wu H Tang S Zhou M Xue J Yu Z Zhu J. Tim-3 suppresses autoimmune hepatitis via the p38/MKP-1 pathway in Th17 cells. FEBS Open Bio. (2021) 11:1406–16. doi: 10.1002/2211-5463.13148, PMID: 33728805 PMC8091815

[131] Klaeschen AS Wolf D Brossart P Bieber T Wenzel J. JAK inhibitor ruxolitinib inhibits the expression of cytokines characteristic of cutaneous lupus erythematosus. Exp Dermatol. (2017) 26:728–30. doi: 10.1111/exd.13253, PMID: 27892610

[132] Hsu L Armstrong AW. JAK inhibitors: treatment efficacy and safety profile in patients with psoriasis. J Immunol Res. (2014) 2014:283617. doi: 10.1155/2014/283617, PMID: 24883332 PMC4027021

[133] Dang C Lu Y Chen X Li Q. Baricitinib ameliorates experimental autoimmune encephalomyelitis by modulating the Janus kinase/signal transducer and activator of transcription signaling pathway. Front Immunol. (2021) 12:650708. doi: 10.3389/fimmu.2021.650708, PMID: 33927721 PMC8076548

[134] Papp K Menter M Raman M Disch D Schlichting D Gaich C. A randomized phase 2b trial of baricitinib, an oral Janus kinase (JAK) 1/JAK2 inhibitor, in patients with moderate-to-severe psoriasis. Br J Dermatol. (2016) 174:1266–76. doi: 10.1111/bjd.14403, PMID: 26800231

[135] Staniszewska M Kiełbowski K Rusińska K Bakinowska E Gromowska E Pawlik A. Targeting cyclin-dependent kinases in rheumatoid arthritis and psoriasis–a review of current evidence. Expert Opin Ther Targets. (2023) 27:1097–113. doi: 10.1080/14728222.2023.2285784, PMID: 37982244

[136] Hong H Zeng Y Jian W Li L Lin L Mo Y. CDK 7 inhibition suppresses rheumatoid arthritis inflammation via blockage of NF-κB activation and IL-1β/IL-6 secretion. J Cell Mol Med. (2018) 22:1292–301. doi: 10.1111/jcmm.13414, PMID: 29083085 PMC5783872

[137] Xia Y Lin L-Y Liu M-L Wang Z Hong H-H Guo X-G. Selective inhibition of CDK7 ameliorates experimental arthritis in mice. Clin Exp Med. (2015) 15:269–75. doi: 10.1007/s10238-014-0305-6, PMID: 25149277

[138] Zhang L Pitcher LE Prahalad V Niedernhofer LJ Robbins PD. Targeting cellular senescence with senotherapeutics: senolytics and senomorphics. FEBS J. (2023) 290:1362–83. doi: 10.1111/febs.16350, PMID: 35015337

[139] Fei F Yu Y Schmitt A Rojewski MT Chen B Götz M. Dasatinib inhibits the proliferation and function of CD4+ CD25+ regulatory T cells. Br J haematol. (2009) 144:195–205. doi: 10.1111/j.1365-2141.2008.07433.x, PMID: 19016717

[140] Min HK Kim SH Won JY Kim KW Lee JY Lee SH. Dasatinib, a selective tyrosine kinase inhibitor, prevents joint destruction in rheumatoid arthritis animal model. Int J Rheumatic Diseases. (2023) 26:718–26. doi: 10.1111/1756-185X.14627, PMID: 36808837

[141] Azizi G Goudarzvand M Afraei S Sedaghat R Mirshafiey A. Therapeutic effects of dasatinib in mouse model of multiple sclerosis. Immunopharmacol Immunotoxicol. (2015) 37:287–94. doi: 10.3109/08923973.2015.1028074, PMID: 25975582

[142] ul Islam B Suhail M MS K Ahmad A Zughaibi TA Husain FM. Flavonoids and PI3K/Akt/mTOR signaling cascade: a potential crosstalk in anticancer treatment. Curr Medicinal Chem. (2021) 28:8083–97. doi: 10.2174/0929867328666210804091548, PMID: 34348607

[143] Dos Santos M Poletti PT Favero G Stacchiotti A Bonomini F Montanari CC. Protective effects of quercetin treatment in a pristane-induced mouse model of lupus nephritis. Autoimmunity. (2018) 51:69–80. doi: 10.1080/08916934.2018.1442828, PMID: 29480020

[144] Xiong F Shen K Long D Zhou S Ruan P Xin Y. Quercetin ameliorates lupus symptoms by promoting the apoptosis of senescent Tfh cells via the Bcl-2 pathway. Immun Ageing. (2024) 21:69. doi: 10.1186/s12979-024-00474-9, PMID: 39407236 PMC11476537

[145] Yosef R Pilpel N Tokarsky-Amiel R Biran A Ovadya Y Cohen S. Directed elimination of senescent cells by inhibition of BCL-W and BCL-XL. Nat Commun. (2016) 7:11190. doi: 10.1038/ncomms11190, PMID: 27048913 PMC4823827

[146] Fuhrmann-Stroissnigg H Niedernhofer LJ Robbins PD. Hsp90 inhibitors as senolytic drugs to extend healthy aging. Cell Cycle. (2018) 17:1048–55. doi: 10.1080/15384101.2018.1475828, PMID: 29886783 PMC6110594

[147] Lanneau D Brunet M Frisan E Solary E Fontenay M Garrido C. Heat shock proteins: essential proteins for apoptosis regulation. J Cell Mol Med. (2008) 12:743–61. doi: 10.1111/j.1582-4934.2008.00273.x, PMID: 18266962 PMC4401125

[148] Georgopoulos C McFarland H. Heat shock proteins in multiple sclerosis and other autoimmune diseases. Immunol Today. (1993) 14:373–5. doi: 10.1016/0167-5699(93)90135-8, PMID: 8397775

[149] Stevens T Smith S Rampton D. Antibodies to human recombinant lipocortin-L in inflammatory bowel disease. Clin Sci. (1993) 84:381–6. doi: 10.1042/cs0840381, PMID: 8097682

[150] Hu S Xu Q Xiao W Huang M. The expression of molecular chaperone HSP90 and IL-6 in patients with systemic lupus erythematosus. J Huazhong Univ Sci Technol. (2006) 26:664–6. doi: 10.1007/s11596-006-0609-1, PMID: 17357483

[151] Rufini A Tucci P Celardo I Melino G. Senescence and aging: the critical roles of p53. Oncogene. (2013) 32:5129–43. doi: 10.1038/onc.2012.640, PMID: 23416979

[152] Takatori H Kawashima H Suzuki K Nakajima H. Role of p53 in systemic autoimmune diseases. Crit Reviews™ Immunol. (2014) 34:509–16. doi: 10.1615/CritRevImmunol.2014012193, PMID: 25597313

[153] Han Z Boyle DL Shi Y Green DR Firestein GS. Dominant-negative p53 mutations in rheumatoid arthritis. Arthritis Rheumatism. (1999) 42:1088–92. doi: 10.1002/1529-0131(199906)42:6<1088::AID-ANR4>3.0.CO;2-E 10366100

[154] Chung H Kim C. Nutlin-3a for age-related macular degeneration. Aging (Albany NY). (2022) 14:5614. doi: 10.18632/aging.204187, PMID: 35849498 PMC9365563

[155] Elsallabi O Patruno A Pesce M Cataldi A Carradori S Gallorini M. Fisetin as a senotherapeutic agent: biopharmaceutical properties and crosstalk between cell senescence and neuroprotection. Molecules. (2022) 27:738. doi: 10.3390/molecules27030738, PMID: 35164003 PMC8839434

[156] Hashimoto M Yamamoto S Iwasa K Yamashina K Ishikawa M Maruyama K. The flavonoid Baicalein attenuates cuprizone-induced demyelination via suppression of neuroinflammation. Brain Res bulletin. (2017) 135:47–52. doi: 10.1016/j.brainresbull.2017.09.007, PMID: 28923306 PMC5700834

[157] Dryden GW Lam A Beatty K Qazzaz HH McClain CJ. A pilot study to evaluate the safety and efficacy of an oral dose of (–)-epigallocatechin-3-gallate–rich Polyphenon E in patients with mild to moderate ulcerative colitis. Inflammatory bowel diseases. (2013) 19:1904–12. doi: 10.1097/MIB.0b013e31828f5198, PMID: 23846486

[158] Yang Y Gocke AR Lovett-Racke A Drew PD Racke MK. PPAR alpha regulation of the immune response and autoimmune encephalomyelitis. PPAR Res. (2008) 2008:546753. doi: 10.1155/2008/546753, PMID: 18645614 PMC2465123

[159] Malumbres M. Cyclin-dependent kinases. Genome Biol. (2014) 15:1–10. doi: 10.1186/gb4184, PMID: 25180339 PMC4097832

[160] Komatsu R Fujii R Ogasawara T Suzuki-Takahashi Y Chen S Sugishita Y. CDK6-Dependent, CDK4-independent synovial hyperplasia in arthritic mice and tumor necrosis factor-α-induced proliferation of synovial fibroblasts. Int J Mol Sci. (2017) 26(3):1151. doi: 10.3390/ijms26031151, PMID: 39940918 PMC11817658

[161] Staniszewska M Kiełbowski K Rusińska K Bakinowska E Gromowska E Pawlik A. Targeting cyclin-dependent kinases in rheumatoid arthritis and psoriasis–a review of current evidence. Expert Opin Ther Targets. (2023) 27:1097–113. doi: 10.1080/14728222.2023.2285784, PMID: 37982244

[162] Gartel AL Serfas MS Tyner AL. p21—negative regulator of the cell cycle. Proc Soc Exp Biol Med. (1996) 213:138–49. doi: 10.3181/00379727-213-44046, PMID: 8931660

[163] Kim U Kim KS Park J-K Um H-D. Involvement of the p53/p21 complex in p53-dependent gene expression. Biochem Biophys Res Commun. (2022) 621:151–6. doi: 10.1016/j.bbrc.2022.07.022, PMID: 35834924

[164] Perlman H Bradley K Liu H Cole S Shamiyeh E Smith RC. IL-6 and matrix metalloproteinase-1 are regulated by the cyclin-dependent kinase inhibitor p21 in synovial fibroblasts. J Immunol. (2003) 170:838–45. doi: 10.4049/jimmunol.170.2.838, PMID: 12517948

[165] Tasaki D Tsuruda K Sun S Tsumura Y Asano S Suzuki Y. A double-blind, placebo-controlled, randomized multiple dose phase 1b trial of a CDK4/6 inhibitor, TCK-276, in patients with active rheumatoid arthritis. Rheumatology. (2024) 64(3):1036–44. doi: 10.1093/rheumatology/keae357, PMID: 39002122

[166] Lin Y Xue K Li Q Liu Z Zhu Z Chen J. Cyclin-dependent kinase 7 promotes Th17/Th1 cell differentiation in psoriasis by modulating glycolytic metabolism. J Invest Dermatol. (2021) 141:2656–67. e11. doi: 10.1016/j.jid.2021.04.018, PMID: 34004188

[167] Schmitz F Heit A Dreher S Eisenächer K Mages J Haas T. Mammalian target of rapamycin (mTOR) orchestrates the defense program of innate immune cells. Eur J Immunol. (2008) 38:2981–92. doi: 10.1002/eji.200838761, PMID: 18924132

[168] Blagosklonny MV. Cell senescence, rapamycin and hyperfunction theory of aging. Cell Cycle. (2022) 21:1456–67. doi: 10.1080/15384101.2022.2054636, PMID: 35358003 PMC9278457

[169] Asghari F Karimi M Pourfathollah A. mTORC1 inhibition may improve T lymphocytes affected by aging. Immunopharmacol Immunotoxicol. (2023) 45:719–29. doi: 10.1080/08923973.2023.2232101, PMID: 37581412

[170] Wang R Sunchu B Perez VI. Rapamycin and the inhibition of the secretory phenotype. Exp gerontol. (2017) 94:89–92. doi: 10.1016/j.exger.2017.01.026, PMID: 28167236

[171] Caza T Wijewardena C Al-Rabadi L Perl A. Cell type-specific mechanistic target of rapamycin-dependent distortion of autophagy pathways in lupus nephritis. Trans Res. (2022) 245:55–81. doi: 10.1016/j.trsl.2022.03.004, PMID: 35288362 PMC9240418

[172] Li Z Chen L Sun Y Li G. Rapamycin relieves inflammation of experimental autoimmune encephalomyelitis by altering the balance of Treg/Th17 in a mouse model. Neurosci letters. (2019) 705:39–45. doi: 10.1016/j.neulet.2019.04.035, PMID: 31004709

[173] Zhang J Wang X Wang R Chen G Wang J Feng J. Rapamycin treatment alleviates chronic GVHD-induced lupus nephritis in mice by recovering IL-2 production and regulatory T cells while inhibiting effector T cells activation. Biomedicines. (2023) 11:949. doi: 10.3390/biomedicines11030949, PMID: 36979928 PMC10045991

[174] Zhang S Hu X Su Q Zhang H Cheng T Wang J. Rapamycin suppresses rheumatoid arthritis fibroblast synovial cell proliferation and induces apoptosis via the AKT/mTORC1 pathway. Rheumatol Autoimmunity. (2024) 4:156–64. doi: 10.1002/rai2.12120

[175] Tang Y Bai Z Qi J Lu Z Wang G Jin M. Altered peripheral B lymphocyte homeostasis and functions mediated by IL-27 via activating the mammalian target of rapamycin signaling pathway in patients with rheumatoid arthritis. Clin Exp Immunol. (2021) 206:354–65. doi: 10.1111/cei.13663, PMID: 34558072 PMC8561691

[176] Kim J-W Choe J-Y Park S-H. Metformin and its therapeutic applications in autoimmune inflammatory rheumatic disease. Korean J Internal Med. (2021) 37:13. doi: 10.3904/kjim.2021.363, PMID: 34879473 PMC8747910

[177] Dziedzic A Saluk-Bijak J Miller E Bijak M. Metformin as a potential agent in the treatment of multiple sclerosis. Int J Mol Sci. (2020) 21:5957. doi: 10.3390/ijms21175957, PMID: 32825027 PMC7503488

[178] Gharib M Elbaz W Darweesh E Sabri NA Shawki MA. Efficacy and safety of metformin use in rheumatoid arthritis: a randomized controlled study. Front Pharmacol. (2021) 12:726490. doi: 10.3389/fphar.2021.726490, PMID: 34630103 PMC8493211

[179] Teng X Brown J Morel L. Redox homeostasis involvement in the pharmacological effects of metformin in systemic lupus erythematosus. Antioxidants Redox Signaling. (2022) 36:462–79. doi: 10.1089/ars.2021.0070, PMID: 34619975 PMC8982129

[180] Bharath LP Nikolajczyk BS. The intersection of metformin and inflammation. Am J Physiology-Cell Physiol. (2021) 320:C873–C9. doi: 10.1152/ajpcell.00604.2020, PMID: 33689478 PMC8163577

[181] Wang Y Jia X Cong B. Advances in the mechanism of metformin with wide-ranging effects on regulation of the intestinal microbiota. Front Microbiol. (2024) 15:1396031. doi: 10.3389/fmicb.2024.1396031, PMID: 38855769 PMC11157079

[182] Oliveira A Monteiro VVS Navegantes-Lima KC Reis JF Gomes R Rodrigues DVS. Resveratrol role in autoimmune disease—a mini-review. Nutrients. (2017) 9:1306. doi: 10.3390/nu9121306, PMID: 29194364 PMC5748756

[183] Frémont L. Biological effects of resveratrol. Life Sci. (2000) 66:663–73. doi: 10.1016/S0024-3205(99)00410-5, PMID: 10680575

[184] Zequan C Guowei X Jian A. Resveratrol attenuates rheumatoid arthritis induce neutrophil extracellular traps via TLR-4 mediated inflammation in C57BL/6 mice. Physiol Res. (2024) 73:91–104. doi: 10.33549/physiolres.935172, PMID: 38466008 PMC11019621

[185] Samy DM Zaki EI Hassaan PS Abdelmonsif DA Mohamed DY Saleh SR. Neurobehavioral, biochemical and histological assessment of the effects of resveratrol on cuprizone-induced demyelination in mice: role of autophagy modulation. J Physiol Biochem. (2023) 79:583–96. doi: 10.1007/s13105-023-00959-z, PMID: 37131098 PMC10338563

[186] Yildiz G Yildiz Y A Ulutas P Yaylali A Ural M. Resveratrol pretreatment ameliorates TNBS colitis in rats. Recent Patents Endocrine Metab Immune Drug Discovery. (2015) 9:134–40. doi: 10.2174/1872214809666150806105737, PMID: 26246013 PMC4997944

[187] Hussain M Javeed A Ashraf M Zhao Y Mukhtar MM Rehman MU. Aspirin and immune system. Int immunopharmacol. (2012) 12:10–20. doi: 10.1016/j.intimp.2011.11.021, PMID: 22172645

[188] Schett G Tohidast-Akrad M Smolen JS Schmid BJ Steiner CW Bitzan P. Activation, differential localization, and regulation of the stress-activated protein kinases, extracellular signal–regulated kinase, c-Jun N-terminal kinase, and p38 mitogen-activated protein kinase, in synovial tissue and cells in rheumatoid arthritis. Arthritis Rheumatism. (2000) 43:2501–12. doi: 10.1002/1529-0131(200011)43:11<2501::AID-ANR18>3.0.CO;2-K, PMID: 11083274

[189] Waetzig GH Seegert D Rosenstiel P Nikolaus S Schreiber S. p38 mitogen-activated protein kinase is activated and linked to TNF-α signaling in inflammatory bowel disease. J Immunol. (2002) 168:5342–51. doi: 10.4049/jimmunol.168.10.5342, PMID: 11994493

[190] Mavropoulos A Orfanidou T Liaskos C Smyk DS Billinis C Blank M. p38 mitogen-activated protein kinase (p38 MAPK)-mediated autoimmunity: lessons to learn from ANCA vasculitis and pemphigus vulgaris. Autoimmun Rev. (2013) 12:580–90. doi: 10.1016/j.autrev.2012.10.019, PMID: 23207287

[191] Xue C Yao Q Gu X Shi Q Yuan X Chu Q. Evolving cognition of the JAK-STAT signaling pathway: autoimmune disorders and cancer. Signal transduction targeted Ther. (2023) 8:204. doi: 10.1038/s41392-023-01468-7, PMID: 37208335 PMC10196327

[192] Xu M Tchkonia T Kirkland JL. Perspective: Targeting the JAK/STAT pathway to fight age-related dysfunction. Pharmacol Res. (2016) 111:152–4. doi: 10.1016/j.phrs.2016.05.015, PMID: 27241018 PMC5026572

[193] Hosseini A Gharibi T Mohammadzadeh A Ebrahimi-Kalan A Jadidi-Niaragh F Babaloo Z. Ruxolitinib attenuates experimental autoimmune encephalomyelitis (EAE) development as animal models of multiple sclerosis (MS). Life Sci. (2021) 276:119395. doi: 10.1016/j.lfs.2021.119395, PMID: 33781828

[194] Park JJ Little AJ Vesely MD. Treatment of cutaneous lupus with topical ruxolitinib cream. JAAD Case Rep. (2022) 28:133–5. doi: 10.1016/j.jdcr.2022.08.038, PMID: 36159722 PMC9494033

[195] Smolen JS Landewé RB Bergstra SA Kerschbaumer A Sepriano A Aletaha D. EULAR recommendations for the management of rheumatoid arthritis with synthetic and biological disease-modifying antirheumatic drugs: 2022 update. Ann rheumatic diseases. (2023) 82:3–18. doi: 10.1136/ard-2022-223356, PMID: 36357155

[196] Hafeez U Gan HK Scott AM. Monoclonal antibodies as immunomodulatory therapy against cancer and autoimmune diseases. Curr Opin Pharmacol. (2018) 41:114–21. doi: 10.1016/j.coph.2018.05.010, PMID: 29883853

[197] Wu H Deng Y Feng Y Long D Ma K Wang X. Epigenetic regulation in B-cell maturation and its dysregulation in autoimmunity. Cell Mol Immunol. (2018) 15:676–84. doi: 10.1038/cmi.2017.133, PMID: 29375128 PMC6123482

[198] Furie R Rovin BH Houssiau F Malvar A Teng YO Contreras G. Two-year, randomized, controlled trial of belimumab in lupus nephritis. New Engl J Med. (2020) 383:1117–28. doi: 10.1056/NEJMoa2001180, PMID: 32937045

[199] Almaani S Rovin BH. B-cell therapy in lupus nephritis: an overview. Nephrol Dialysis Transplantation. (2019) 34:22–9. doi: 10.1093/ndt/gfy267, PMID: 30165690 PMC6934028

[200] Gomez A Jägerback S Sjöwall C Parodis I. Belimumab and antimalarials combined against renal flares in patients treated for extra-renal systemic lupus erythematosus: results from 4 phase III clinical trials. Rheumatology. (2024) 63:338–48. doi: 10.1093/rheumatology/kead253, PMID: 37228028 PMC10836979

[201] Sans-Pola C Danés I Bosch JÀ Marrero-Álvarez P Cortés J Agustí A. Off-label use of rituximab in patients with systemic lupus erythematosus with extrarenal disease activity: a retrospective study and literature review. Front Med. (2023) 10:1159794. doi: 10.3389/fmed.2023.1159794, PMID: 37305139 PMC10248418

[202] Dass S Vital EM Emery P. Rituximab: B-cell depletion therapy for the treatment of rheumatoid arthritis. Int J Clin Rheumatol. (2006) 1:293. doi: 10.2217/17460816.1.3.293 17150009

[203] Ho C Spry C. Rituximab for Granulomatosis with Polyangiitis or Microscopic Polyangiitis: A Review of the Clinical effectiveness, Cost-effectiveness, and Guidelines. CADTH rapid response report: summary with critical appraisal chuong ho and lorna adcock. (Ottawa (ON): Canadian Agency for Drugs and Technologies in Health) (2018). 29470031

[204] Frasca D Diaz A Romero M Garcia D Blomberg BB. B cell immunosenescence. Annu Rev Cell Dev Biol. (2020) 36:551–74. doi: 10.1146/annurev-cellbio-011620-034148, PMID: 33021823 PMC8060858

[205] Nakken B Munthe LA Konttinen YT Sandberg AK Szekanecz Z Alex P. B-cells and their targeting in rheumatoid arthritis—current concepts and future perspectives. Autoimmun Rev. (2011) 11:28–34. doi: 10.1016/j.autrev.2011.06.010, PMID: 21777703

[206] Montalban X Matthews PM Simpson A Petrie JL Sammon C Ramagopalan S. Real-world evaluation of ocrelizumab in multiple sclerosis: A systematic review. Ann Clin Trans neurol. (2023) 10:302–11. doi: 10.1002/acn3.51732, PMID: 36728340 PMC10014013

[207] Taylor PC Quattrocchi E Mallett S Kurrasch R Petersen J Chang DJ. Ofatumumab, a fully human anti-CD20 monoclonal antibody, in biological-naive, rheumatoid arthritis patients with an inadequate response to methotrexate: a randomised, double-blind, placebo-controlled clinical trial. Ann rheumatic diseases. (2011) 70:2119–25. doi: 10.1136/ard.2011.151522, PMID: 21859685 PMC3212699

[208] Hara K Togashi M. Successful treatment of multiple sclerosis with refractory rheumatoid arthritis using ofatumumab: A case report. Clin Exp Neuroimmunol. (2024) 15:122–5. doi: 10.1111/cen3.12780

[209] Lin TS. Ofatumumab: a novel monoclonal anti-CD20 antibody. Pharmacogenomics personalized Med. (2010) 3:51–9. doi: 10.2147/PGPM.S6840, PMID: 23226042 PMC3513208

[210] Rovin BH Furie RA Ross Terres JA Giang S Schindler T Turchetta A. Kidney outcomes and preservation of kidney function with obinutuzumab in patients with lupus nephritis: A *post hoc* analysis of the NOBILITY Trial. Arthritis Rheumatol. (2024) 76:247–54. doi: 10.1002/art.42734, PMID: 37947366

[211] Martin S-J Guenette M Oh J. Evaluating the therapeutic potential of ublituximab in the treatment of MS: design, development and place in therapy. Drug Design Dev Ther. (2024) 18:3025–42. doi: 10.2147/DDDT.S388410, PMID: 39050801 PMC11268567

[212] Gómez-Urquiza JL Romero-Bejar JL Chami-Peña S Suleiman-Martos N Cañadas-De la Fuente GA Molina E. Efficacy and safety of new B cell-targeted biologic agent for the treatment of systemic lupus erythematosus: A systematic review and meta-analysis. J Clin Med. (2023) 12:4848. doi: 10.3390/jcm12144848, PMID: 37510963 PMC10382055

[213] Rossi EA Chang C-H Goldenberg DM. Anti-CD22/CD20 Bispecific antibody with enhanced trogocytosis for treatment of Lupus. PloS One. (2014) 9:e98315. doi: 10.1371/journal.pone.0098315, PMID: 24841238 PMC4026529

[214] Kaegi C Steiner UC Wuest B Crowley C Boyman O. Systematic review of safety and efficacy of atacicept in treating immune-mediated disorders. Front Immunol. (2020) 11:433. doi: 10.3389/fimmu.2020.00433, PMID: 32265917 PMC7105675

[215] Lafayette R Barbour S Israni R Wei X Eren N Floege J. A phase 2b, randomized, double-blind, placebo-controlled, clinical trial of atacicept for treatment of IgA nephropathy. Kidney Int. (2024) 105:1306–15. doi: 10.1016/j.kint.2024.03.012, PMID: 38552841

[216] Langley RG Sofen H Dei-Cas I Reich K Sigurgeirsson B Warren RB. Secukinumab long-term efficacy and safety in psoriasis through to year 5 of treatment: results of a randomized extension of the phase III ERASURE and FIXTURE trials. Br J Dermatol. (2023) 188:198–207. doi: 10.1093/bjd/ljac040, PMID: 36763857

[217] Wang Y Xiao Y Zhang L Li F Hu H Peng X. Superior effect of adalimumab versus secukinumab on ultrasound-confirmed synovitis in psoriatic arthritis: comprehensive evidence from musculoskeletal ultrasound and clinical assessments. J Dermatol Treat. (2024) 35:2411849. doi: 10.1080/09546634.2024.2411849, PMID: 39370135

[218] Ruwaard J l’Ami M Kneepkens E Krieckaert C Nurmohamed M Hooijberg F. Interval prolongation of etanercept in rheumatoid arthritis, ankylosing spondylitis, and psoriatic arthritis: a randomized controlled trial. Scandinavian J Rheumatol. (2023) 52:129–36. doi: 10.1080/03009742.2022.2028364, PMID: 35234569

[219] Li C Sunhe Y Zhou H Dong W. Efficacy and safety evaluations of adalimumab biosimilars in the treatment of psoriasis. J Dermatol Treat. (2023) 34:2249145. doi: 10.1080/09546634.2023.2249145, PMID: 37608703

[220] Kounatidis DC Papadimitropoulos V Avramidis K Plenga E Tsiara I Avgoustou E. Pneumocystosis in a patient with rheumatoid arthritis on adalimumab therapy: A case-based review. Rheumatol Int. (2024) 44:363–7. doi: 10.1007/s00296-023-05483-3, PMID: 37851077

[221] Azadbakht S Seighali M Azadbakht S Azadbakht M. Effectiveness of adalimumab in severe ulcerative colitis: A systematic review and a meta-analysis. Health Sci Rep. (2024) 7:e2210. doi: 10.1002/hsr2.2210, PMID: 39035679 PMC11258201

[222] Pugliese D Felice C Papa A Gasbarrini A Rapaccini GL Guidi L. Anti TNF-α therapy for ulcerative colitis: current status and prospects for the future. Expert Rev Clin Immunol. (2017) 13:223–33. doi: 10.1080/1744666X.2017.1243468, PMID: 27687496

[223] Sukhanova AM Gilavian MA Melnik EV Shikh EV Petukhov AE Gegechkori VI. An overview of adalimumab therapy for ankylosing spondylitis. Curr Rheumatol Rev. (2024) 20:501–13. doi: 10.2174/0115733971289295240223095751, PMID: 38415452 PMC11340288

[224] Oddis CV Rockette HE Zhu L Koontz DC Lacomis D Venturupalli S. Randomized trial of tocilizumab in the treatment of refractory adult polymyositis and dermatomyositis. ACR Open Rheumatol. (2022) 4:983–90. doi: 10.1002/acr2.11493, PMID: 36128663 PMC9661830

[225] Unizony S Matza MA Jarvie A O’Dea D Fernandes AD Stone JH. Treatment for giant cell arteritis with 8 weeks of prednisone in combination with tocilizumab: a single-arm, open-label, proof-of-concept study. Lancet Rheumatol. (2023) 5:e736–e42. doi: 10.1016/S2665-9913(23)00265-5, PMID: 38251564

[226] Doctor M Murthy SI Rajasekhar L. Tocilizumab in recalcitrant bilateral scleritis in a case of relapsing polychondritis: A 17-year follow up. Ocular Immunol Inflammation. (2023) 31:870–3. doi: 10.1080/09273948.2022.2058555, PMID: 35695904

[227] Shahada Z Kudsi M. Successfully treated refractory Sjögren’s syndrome myelopathy with tocilizumab; case report. IJS Global Health. (2024) 7:e00470. doi: 10.1097/GH9.0000000000000470

[228] Sun A Wang X Qu J Wu Y. The efficacy and safety of intravenous tocilizumab to treat Graves’ ophthalmopathy: A systematic review and single-arm meta-analysis. J Clin Endocrinol Metab. (2024) 110:e886–96. doi: 10.1210/clinem/dgae711, PMID: 39401327

[229] Özdemir B Latifyan S Perreau M Fenwick C Alberio L Waeber G. Cytokine-directed therapy with tocilizumab for immune checkpoint inhibitor-related hemophagocytic lymphohistiocytosis. Ann Oncol. (2020) 31:1775–8. doi: 10.1016/j.annonc.2020.08.2101, PMID: 32858151

[230] Johnson E Lesh N Hildebrand G Shah H Osman A Abou-Ismail MY. Bortezomib for the management of relapsed or refractory warm autoimmune hemolytic anemia: A scoping review. Blood. (2021) 138:4149. doi: 10.1182/blood-2021-153437

[231] Truong TM Pathak GN Singal A Taranto V Rao BK. Deucravacitinib: the first FDA-approved oral TYK2 inhibitor for moderate to severe plaque psoriasis. Ann Pharmacother. (2024) 58:416–27. doi: 10.1177/10600280231153863, PMID: 37341177

[232] Schett G Feldman T Abramson J Hu B Müller F Koegel A. P105 NEX-T CD19 chimeric antigen receptor (CAR) T-cell therapy CC-97540 (BMS-986353): Preclinical and translational evidence of deep B-cell depletion suitable for study in severe refractory autoimmune diseases. Arch Dis childhood. (2024) 11. doi: 10.1136/lupus-2024-el.159

[233] Mackensen A Müller F Mougiakakos D Böltz S Wilhelm A Aigner M. Anti-CD19 CAR T cell therapy for refractory systemic lupus erythematosus. Nat Med. (2022) 28:2124–32. doi: 10.1038/s41591-022-02017-5, PMID: 36109639

[234] Seshadri MR Gupta S Lincoln R Okinishi N Thomas N Wilmoth J. A Study of Kyv-101, a CD19 CAR T cell therapy, in participants with treatment refractory progressive multiple sclerosis. Blood. (2024) 144:3469–1. doi: 10.1182/blood-2024-204169

[235] Kim DK Park JY Kang YJ Khang D. Drug repositioning of metformin encapsulated in PLGA combined with photothermal therapy ameliorates rheumatoid arthritis. Int J Nanomed. (2025) 2023:7267–85. doi: 10.2147/IJN.S438388, PMID: 38090362 PMC10711299

[236] Piquet AL Zekeridou A Nie EH Borie D Chung J Dalakas MC. Design of KYSA-8, A phase 2, open-label, multicenter study of KYV-101, an autologous fully human anti-CD19 chimeric antigen receptor (CAR) T-cell therapy, in treatment refractory stiff-person syndrome (P2-8.018). Neurology. (2025) 104. doi: 10.1212/WNL.0000000000210631

[237] Amor C Feucht J Leibold J Ho Y-J Zhu C Alonso-Curbelo D. Senolytic CAR T cells reverse senescence-associated pathologies. Nature. (2020) 583:127–32. doi: 10.1038/s41586-020-2403-9, PMID: 32555459 PMC7583560

[238] Eskiocak O Chowdhury S Shah V Nnuji-John E Chung C Boyer JA. Senolytic CAR T cells reverse aging-associated defects in intestinal regeneration and fitness. bioRxiv. (2024) 2024.03.19.585779. doi: 10.1101/2024.03.19.585779, PMID: 38529506 PMC10962734

[239] Amor C Fernández-Maestre I Chowdhury S Ho Y-J Nadella S Graham C. Prophylactic and long-lasting efficacy of senolytic CAR T cells against age-related metabolic dysfunction. Nat Aging. (2024) 4:336–49. doi: 10.1038/s43587-023-00560-5, PMID: 38267706 PMC10950785

