# Supplementary material for: Immune cell senescence in autoimmunity: implications for disease pathogenesis and therapeutic targeting
Source: Front Immunol. 2025 Aug 7;16:1596686. doi: 10.3389/fimmu.2025.1596686 (PMC12367673; doi:10.3389/fimmu.2025.1596686)
Supplement: Supplementary file 1 [file Table1.docx]

Supplementary Table 1. Overview of targeted immunotherapies for autoimmune diseases: Monoclonal antibodies and CAR-T cell therapies in clinical development

| **Therapy** | **Targets** | **Targeted Autoimmune Diseases** | **Study title** | **Study phase** | **Status** | **Clinical ID** |
| --- | --- | --- | --- | --- | --- | --- |
| CC-97540 | CD19 | SLE, Idiopathic Inflammatory Myopathy, Systemic Sclerosis | Participants with severe, refractory autoimmune diseases receiving CC-97540, CD-19-Targeted Nex-T CAR-T Cells | Phase I | Recruiting | NCT05869955 |
| KYV-101 | CD19 | Refractory Systemic Lupus Erythematosus | Patients with refractory lupus nephritis were treated with KYSA-1, an anti-CD19 chimeric antigen receptor T-cell (CD19 CAR-T) therapy, as part of the study | Phase I/II | Recruiting | NCT05938725 |
| CABA-201 | CD19 | Systemic Sclerosis | RESET-SSc: An Open-Label Study to Evaluate the Safety and Efficacy of CABA-201, a CD19-CAR T Cell Therapy, in Subjects with Systemic Sclerosis | Phase I/II | Recruiting | NCT06328777 |
| Rituximab | CD20 | RA | A clinical investigation evaluating the efficacy of MabThera (Rituximab) in rheumatoid arthritis patients who did not achieve an adequate response to a single anti-TNF inhibitor therapy | Phase III | Completed | NCT02079532 |
|  |  | SLE | A Study to Evaluate the Safety of Rituximab Retreatment in Subjects With Systemic Lupus Erythematosus | Phase II/III | Completed | NCT00137969 |
|  |  | Nephrotic Syndrome | Rituximab for Idiopathic Nephrotic Syndrome | Phase III | Completed | NCT04494438 |
|  |  | Granulomatosis with polyangiitis | Rituximab Vasculitis Maintenance Study | Phase III | Completed | NCT01697267 |
|  |  | Progressive MS | [Intrathecal Rituximab in Progressive Multiple Sclerosis](https://clinicaltrials.gov/study/NCT02545959?cond=Rituximab&page=11&rank=106) | Phase II | Completed | NCT02545959 |
| Veltuzumab | CD20 | RA | Subjects with moderate to severe rheumatoid arthritis in the VELVET dose range-finding trial of Veltuzumab. | Phase II | Terminated | NCT01390545 |
| Ocrelizumab | CD20 | RA | The study included individuals diagnosed with moderate to severe rheumatoid arthritis (RA) who were evaluated for treatment with Ocrelizumab. | Phase I/II | Terminated | NCT02720120 |
|  |  | RA | The study assessed the efficacy, safety, and pharmacokinetic/pharmacodynamic (PK/PD) profile of Ocrelizumab in patients diagnosed with RA | Phase I/II | Terminated | NCT00779220 |
|  |  | RA | The study involved patients with active rheumatoid arthritis who were not achieving sufficient responses to etanercept or adalimumab. These participants were assessed in a comparative analysis of Ocrelizumab combined with methotrexate versus infliximab paired with methotrexate | Phase II | Terminated | NCT00808210 |
|  |  | RA | Subjects with rheumatoid arthritis in a study evaluating the safety of escalating doses of Ocrelizumab. | Phase I/II | Completed | NCT00077870 |
|  |  | RA | In the STAGE study, patients with active rheumatoid arthritis who maintained methotrexate therapy were evaluated, comparing the effects of Ocrelizumab against a placebo. | Phase III | Terminated | NCT00406419 |
|  |  | RA | The FILM study included patients with rheumatoid arthritis who had not previously received methotrexate. These participants were assessed for the efficacy of Ocrelizumab when used in combination with methotrexate | Phase III | Terminated | NCT00485589 |
|  |  | RA | The SCRIPT study involved patients with active rheumatoid arthritis who did not respond to anti-TNF-α therapy. These individuals were evaluated in a comparison of Ocrelizumab versus placebo | Phase III | Terminated | NCT00476996 |
|  |  | RA | Patients with rheumatoid arthritis, showing an inadequate response to methotrexate therapy, in a study evaluating Ocrelizumab compared with placebo. | Phase III | Terminated | NCT00673920 |
|  |  | SLE | The study included patients diagnosed with active systemic lupus erythematosus, who were assessed using two different doses of Ocrelizumab | Phase III | Terminated | NCT00539838 |
|  |  | SLE | Patients with nephritis due to systemic lupus erythematosus in the BELONG study, evaluating Ocrelizumab. | Phase III | Terminated | NCT00626197 |
|  |  | Autoimmune Encephalitis | Efficacy of Ocrelizumab in Autoimmune Encephalitis | Phase II | Terminated | NCT03835728 |
|  |  | Relapsing-Remitting MS | Patients with relapsing-remitting MS in a study evaluating the efficacy and safety of Ocrelizumab. | Phase II | Completed | NCT00676715 |
|  |  | Relapsing-Remitting MS | Participants with relapsing MS in a study comparing Ocrelizumab with IFN-β-1a (Rebif). | Phase III | Completed | NCT01247324 |
|  |  | Relapsing-Remitting MS | Participants with relapsing MS in a study comparing Ocrelizumab to IFN-β-1a (Rebif). | Phase III | Completed | NCT01412333 |
|  |  | Primary Progressive MS | Participants with primary progressive MS in a study evaluating Ocrelizumab. | Phase III | Completed | NCT01194570 |
|  |  | Relapsing-Remitting MS | Participants with early-stage relapsing-remitting MS in a study evaluating the effectiveness and safety of Ocrelizumab. | Phase III | Completed | NCT03085810 |
|  |  | Relapsing-Remitting MS | Participants with relapsing forms of MS in a study evaluating the effects of Ocrelizumab on immune responses. | Phase III | Completed | NCT02545868 |
|  |  | Relapsing-Remitting MS | Patients with relapsing-remitting MS (RRMS) who have had a suboptimal response to an adequate course of disease-modifying treatment (DMT) in a study evaluating Ocrelizumab. | Phase III | Completed | NCT02637856 |
|  |  | Relapsing-Remitting MS | Patients with relapsing-remitting MS (RRMS) who have had a suboptimal response to an adequate course of disease-modifying treatment (DMT) in a study of Ocrelizumab. | Phase III | Completed | NCT02637856 |
|  |  | Relapsing-Remitting MS | A study comparing Rituximab and Ocrelizumab in patients with relapsing-remitting MS (RRMS) (TRIO). | Phase III | Recruiting | NCT05758831 |
|  |  | Relapsing-Remitting MS | A study comparing B cell-tailored Ocrelizumab and standard Ocrelizumab in patients with relapsing-remitting MS (RRMS) (BLOOMS). | Phase IV | Recruiting | NCT05296161 |
|  |  | Relapsing-Remitting MS | A study evaluating Ocrelizumab discontinuation in patients with relapsing MS (AMS05). | Phase IV | Recruiting | NCT05285891 |
| Ofatumumab | CD20 | RA | A Phase I/II study of HuMax-CD20 in patients with active RA. | Phase II | Completed | NCT00291928 |
|  |  | RA | Repeated Ofatumumab treatment courses were evaluated in Trial Hx-CD20-403 for long-term efficacy and safety in RA patients previously administered Ofatumumab or placebo. | Phase II | Terminated | NCT00655824 |
|  |  | RA | Investigation of Ofatumumab clinical efficacy in adult RA patients with inadequate response to MTX therapy. | Phase III | Terminated | NCT00611455 |
|  |  | RA | RA patients with inadequate response to TNF-α antagonist therapy, investigated for clinical efficacy of Ofatumumab. | Phase III | Terminated | NCT00603525 |
|  |  | RA | Patients with RA evaluated for the SC route of administration of Ofatumumab. | Phase I | Completed | NCT00686868 |
|  |  | Relapsing-Remitting MS | Dose-finding analysis of Ofatumumab in RRMS patients. | Phase II | Completed | NCT00640328 |
|  |  | Relapsing-Remitting MS | Ofatumumab was administered subcutaneously to subjects with relapsing-remitting MS. | Phase II | Completed | NCT01457924 |
|  |  | Relapsing-Remitting MS | Ofatumumab was evaluated for safety and efficacy in comparison to Teriflunomide in patients with relapsing MS. | Phase III | Completed | NCT02792231 |
|  |  | Pemphigus Vulgaris | Subjects with pemphigus vulgaris in a long-term extension study of Ofatumumab. | Phase III | Terminated | NCT02613910 |
| Obinutuzumab | CD20 | Lupus Nephritis | Patients with lupus nephritis (LN) evaluated for the safety and efficacy of Obinutuzumab compared with placebo. | Phase II | Completed | NCT02550652 |
| Ublituximab | CD20 | Relapsing MS | [An Extension of the TG1101-RMS201 Trial](https://clinicaltrials.gov/study/NCT03381170?cond=ublituximab&rank=1) | Phase III | Completed | NCT03381170 |
|  |  | Relapsing MS | ULTIMATE 1 trial assessed the efficacy and safety of Ublituximab in participants with relapsing forms of MS | Phase III | Completed | NCT03277261 |
|  |  | Relapsing MS | A clinical trial, ULTIMATE II, was conducted to evaluate the effectiveness and safety of Ublituximab in individuals with relapsing forms of MS | Phase III | Completed | NCT03277248 |
| Epratuzumab | CD 22 | Systemic Lupus Erythematosus | A clinical trial comparing the efficacy of Epratuzumab against a placebo in patients diagnosed with moderate to severe SLE | Phase III | Completed | NCT01261793 |
| Belimumab | BLyS | Active Lupus Nephritis | Two-Year, Randomized, Controlled Trial of Belimumab in Lupus Nephritis | Phase III | Completed | NCT01639339 |
| Atacicept | BLyS and APRIL | Active Lupus Nephritis | A Phase 3 Randomized, Double-Blind, Placebo-Controlled Multicenter, Multinational Study to Evaluate the Efficacy and Safety of Atacicept in Subjects With Active Lupus Nephritis | Phase III | Suspended | NCT05609812 |
|  |  | IgA nephropathy | Subjects with IgA nephropathy in a Phase 3 study of Atacicept. | Phase III | Recruiting | NCT04716231 |
| Adalimumab | TNF | RA | A head-to-head comparison of Abatacept versus Adalimumab. | Phase III | Completed | NCT00929864 |
|  |  | Crohn's Disease | Subjects with Crohn's disease in a study evaluating the induction of clinical remission using the human anti-TNF monoclonal antibody Adalimumab. | Phase III | Completed | NCT00105300 |
|  |  | Active Ankylosing Spondylitis | Safety and Efficacy of Adalimumab in Patients With Active Ankylosing Spondylitis | Phase III | Completed | NCT00195819 |
|  |  | Acute Ulcerative Colitis | Efficacy and Safety of Adalimumab in Subjects With Moderately to Severely Acute Ulcerative Colitis | Phase III | Completed | NCT00385736 |
| Etanercept | TNF | Chronic Plaque-type Psoriasis | Study to Demonstrate Equivalent Efficacy and to Compare Safety of Biosimilar Etanercept (GP2015) and Enbrel | Phase III | Completed | NCT01891864 |
| Infliximab | TNF | RA | RA patients in the FAKIR study were assessed for pharmacokinetic variability of Infliximab. | Observational | Completed | NCT00840957 |
|  |  | RA | RA patients in the AWARE study were compared for Golimumab intravenous (IV) (Simponi Aria) versus Infliximab (Remicade). | Observational | Completed | NCT02728934 |
|  |  | Psoriasis | Patients with psoriasis in the REALITY study, assessing long-term Infliximab use (P05319). | Observational | Completed | NCT00779675 |
| Tocilizumab | IL-6 | Relapsing polychondritis | Tocilizumab in a single-patient study for treating relapsing polychondritis. | Phase II | Completed | NCT01041248 |
|  |  | Giant Cell Arteritis | Tocilizumab (RoActemra/Actemra) in a study evaluating efficacy and safety in participants with giant cell arteritis (GCA). | Phase III | Completed | NCT01791153 |
|  |  | Graves´ Ophthalmopathy | Patients with Graves' ophthalmopathy (Graves' orbitopathy or thyroid eye disease) treated with Tocilizumab. | Phase III | Completed | NCT01297699 |
|  |  | HIV | IL-6 blockade in treated HIV infection (AIDS 347 study). | Phase I/II | Completed | NCT02049437 |
|  |  | Refractory Polymyositis and Dermatomyositis | Patients with refractory polymyositis and dermatomyositis treated with Tocilizumab. | Phase II | Completed | NCT02043548 |
|  |  | Primary Sjögren's Syndrome. | Efficacy of Tocilizumab in Primary Sjögren's Syndrome. | Phase II/III | Completed | NCT01782235 |
|  |  | Hemophagocytic lymphohistiocytosis | Tocilizumab and Hemophagocytic Lymphohistiocytosis (HLH) | Phase II | Withdrawn | NCT02007239 |
| Secukinumab | IL-17 | Psoriasis | Therapeutic Drug Monitoring of Secukinumab in Psoriasis Patients. (BIOLOPTIM-SE | Phase IV | Completed | NCT04080661 |
|  |  | Psoriasis | Safety and Efficacy of Secukinumab in Mild Psoriasis | Phase II | Completed | NCT03131570 |
|  |  | Psoriasis | Impact of Secukinumab on Clinical and Patient Reported Outcomes in Patients with Psoriasis | Observational | Completed | NCT05513014 |
| Bortezomib | Proteasome | Myasthenia Gravis (generalized) or SLE or RA | Bortezomib therapy for antibody-mediated autoimmune diseases (TAVAB study). | Phase II | Terminated | NCT02102594 |
| Deucravacitinib | TYK2 Inhibitor | Active Psoriatic Arthritis | Participants with active psoriatic arthritis (PsA) who are naïve to biologic disease-modifying anti-rheumatic drugs in a study determining the efficacy and safety of Deucravacitinib compared with placebo. | Phase III | Active, not recruiting | NCT04908202 |
| Baricitinib | JAK Inhibitor | Psoriasis | Patients with moderate to severe psoriasis in a Phase 2b study of Baricitinib. | Phase II | Completed | NCT01490632 |
| ASP015K | JAK Inhibitor | Psoriasis | Subjects with moderate to severe psoriasis in a study exploring the efficacy and safety of ASP015K. | Phase II | Completed | [NCT01096862](http://clinicaltrials.gov/ct2/show/NCT01096862) |
| Tofacitinib | JAK Inhibitor | Psoriasis | Asian subjects with moderate to severe plaque psoriasis in a study evaluating the efficacy and safety of CP-690,550. | Phase II | Completed | NCT01815424 |
|  |  | Psoriasis | Patients with moderate to severe chronic plaque psoriasis in a one-year study evaluating the efficacy and safety of CP-690,550. | Phase II | Completed | NCT01309737 |
|  |  | Psoriasis | Patients with moderate to severe chronic plaque psoriasis in a long-term study evaluating the safety and tolerability of CP-690,550. | Phase II | Terminated | NCT01163253 |
|  |  | Psoriasis | Patients with chronic plaque psoriasis treated with Tofacitinib ointment. | Phase II | Completed | NCT01831466 |
|  |  | Psoriasis | Subjects with moderate to severe chronic plaque psoriasis in a study evaluating the effects and safety of treatment, treatment withdrawal, followed by re-treatment with CP-690,550. | Phase III | Completed | NCT01186744 |
|  |  | Psoriasis | Patients with moderate to severe chronic plaque psoriasis in a one-year study evaluating the effects and safety of CP-690,550. | Phase II | Completed | NCT01276639 |
|  |  | RA | Patients with active RA evaluated for the optimal dose of Tofacitinib to achieve low disease activity (LDA) or clinical remission, measured from clinical and structural perspectives. | Phase III | Completed | NCT02566967 |
|  |  | RA | RA subjects were evaluated in a safety study comparing Tofacitinib TNF-α inhibitors | Phase IV | Completed | NCT02092467 |
|  |  | RA | RA patients assessed with musculoskeletal ultrasound for therapeutic response to Tofacitinib. | Phase IV | Completed | NCT02321930 |
|  |  | RA | RA subjects with inadequate response or intolerance to bDMARDs in a Phase 3 study comparing TLL-018 to Tofacitinib (TARA). | Phase III | Recruiting | NCT06020144 |
| TCK-276 | Cdk4/6 Inhibitor | RA | Patients with RA in a study investigating the safety, tolerability, and pharmacokinetics (PK) of oral doses of TCK-276. | Phase I | Completed | NCT05437419 |
